# Antibacterial activity of lysozyme-chitosan oligosaccharide conjugates (LYZOX) against *Pseudomonas aeruginosa*, *Acinetobacter baumannii* and Methicillin-resistant *Staphylococcus aureus*

**DOI:** 10.1371/journal.pone.0217504

**Published:** 2019-05-28

**Authors:** Hiroaki Saito, Yumi Sakakibara, Ayumi Sakata, Rie Kurashige, Daisuke Murakami, Hiroki Kageshima, Akira Saito, Yasunari Miyazaki

**Affiliations:** 1 Department of Respiratory Medicine, Tokyo Medical and Dental University, Tokyo, Japan; 2 Department of Otorhinolaryngology, Graduate School of Medical Sciences, Kyushu University, Fukuoka, Japan; 3 Wako Filter Technology Co., Ltd, Tokyo, Japan; University of South Carolina, UNITED STATES

## Abstract

The recent emergence of antibiotic-resistant bacteria requires the development of new antibiotics or new agents capable of enhancing antibiotic activity. This study evaluated the antibacterial activity of lysozyme-chitosan oligosaccharide conjugates (LYZOX) against *Pseudomonas aeruginosa*, *Acinetobacter baumannii* and methicillin-resistant *Staphylococcus aureus* (MRSA), which should resolve the problem of antibiotic-resistant bacteria. Bactericidal tests showed that LYZOX killed 50% more *P*. *aeruginosa* (NBRC 13275), *A*. *baumannii* and MRSA than the control treatment after 60 min. In addition, LYZOX was shown to inhibit the growth of *P*. *aeruginosa* (NBRC 13275 and PAO1), *A*. *baumannii* and MRSA better than its components. To elucidate the antibacterial mechanism of LYZOX, we performed cell membrane integrity assays, N-phenyl-1-naphthylamine assays, 2-nitrophenyl β-D-galactopyranoside assays and confocal laser scanning microscopy. These results showed that LYZOX affected bacterial cell walls and increased the permeability of the outer membrane and the plasma membrane. Furthermore, each type of bacteria treated with LYZOX was observed by electron microscopy. Electron micrographs revealed that these bacteria had the morphological features of both lysozyme-treated and chitosan oligosaccharide-treated bacteria and that LYZOX destroyed bacterial cell walls, which caused the release of intracellular contents from cells. An acquired drug resistance test revealed that these bacteria were not able to acquire resistance to LYZOX. The hemolytic toxicity test demonstrated the low hemolytic activity of LYZOX. In conclusion, LYZOX exhibited antibacterial activity and low drug resistance in the presence of *P*. *aeruginosa*, *A*. *baumannii* and MRSA and showed low hemolytic toxicity. LYZOX affected bacterial membranes, leading to membrane disruption and the release of intracellular contents and consequent bacterial cell death. LYZOX may serve as a novel candidate drug that could be used for the control of refractory infections.

## Introduction

The overuse and misuse of antibiotics leads to the spread of antibiotic-resistant bacteria, including *Pseudomonas aeruginosa* (*P*. *aeruginosa*), *Acinetobacter baumannii* (*A*. *baumannii*), and methicillin-resistant *Staphylococcus aureus* (MRSA), which created problems of hospital infections and immunocompromised hosts. *P*. *aeruginosa* and *A*. *baumannii* are quickly becoming multidrug-resistant due to their intrinsic resistance mechanisms. This resistance can lead to life-threatening illnesses, such as bloodstream, urinary tract, pulmonary, and device-related infections [1, 2]. *Staphylococcus aureus*, including MRSA, is also an important pathogen that can cause diverse infections, ranging from relatively minor skin infections to serious and life-threatening infections, such as endocarditis, pneumonia, and sepsis [3]. Few effective antibiotics make it difficult to treat MRSA infections. It is estimated that 10 million people will die from antimicrobial resistance each year starting from the year 2050 if we do not take measures against antimicrobial resistance [4]. Although we should avoid using inappropriate antibiotics, the increasing resistance of bacteria to some drugs requires either the development of new antibiotics or new agents capable of enhancing antibiotic activity.

Lysozyme is the most large and abundant antimicrobial peptide (AMP) in the innate defense of the airway surface [5]. It is unlikely for bacteria to develop resistance to AMPs because AMPs exert antimicrobial activity, primarily through mechanisms involving membrane disruption [6]. By contrast, a major problem of AMPs is instability against heating and proteolytic load. In addition, as lysozyme targets the glycosidic bond between N-acetylglucosamine and N-acetylmuraminic acid in peptidoglycan, its efficacy limited against gram-positive pathogens [7, 8].

Chitosan is a major component of the shells of crustaceans and has biological activities, such as antimicrobial and antitumor effects [9, 10]. The antimicrobial action of chitosan in a wide variety of microorganisms, including gram-positive bacteria, gram-negative bacteria and fungi [9, 10], is well known. Furthermore, chitosan and modified chitosan were reported to have antibacterial activity against antibiotic-resistant bacteria (S1 Table) [11–15]. However, chitosan requires acetic acid or hydrochloric acid at a pH lower than 6 for dissolution, which limits its application [10, 16].

The Maillard reaction is the interaction that occurs during controlled dry heating between the ε-amino groups in proteins and the reducing end carbonyl group in polysaccharides. Maillard-type protein-polysaccharide conjugates showed excellent emulsifying properties superior to conventional commercial emulsifiers, as well as heat stability, solubility and antimicrobial activity [7]. Polysaccharides, such as dextran, galactomannan, xyloglycan, pectin and chitosan, can be used as partner saccharides [7]. After chitosan conjugates with lysozyme via the Maillard reaction, the solubility and the antibacterial activity increase [7, 16, 17]. High molecular weight chitosan (HMC) (10 μL/mL in 20 mM phosphate buffer pH 7.0) killed *Escherichia coli* (*E*. *coli*) to a remaining 20%, whereas HMC-lysozyme conjugates (10 μL/mL in the same buffer) killed *E*. *coli* to a remaining 10% [16]. Previous reports showed that lysozyme-galactomannan conjugates (LGC) had antibacterial activity against gram-positive and gram-negative bacteria and were expected to prevent the development of antibiotic-drug resistance [16, 18, 19]. LGCs were observed to kill 40% of *E*. *coli* when cultures were treated at 37°C for 30 min, whereas lysozyme or galactomannan alone was observed to kill 20% of *E*. *coli* [16]. There have been no reports that have evaluated the antibacterial activity of lysozyme-chitosan conjugates against *P*. *aeruginosa*, *A*. *baumannii* and MRSA.

The aim of this study was to evaluate the antibacterial activity of a lysozyme-chitosan oligosaccharide conjugate (LYZOX) against *P*. *aeruginosa*, *A*. *baumannii* and MRSA. We compared the antibacterial activity of LYZOX with that of LGC, which is another modified lysozyme, and investigated the antibacterial mechanism of LYZOX.

## Materials and methods

### Materials

*P*. *aeruginosa* (NBRC 13275 and PAO1) was purchased from Biological Resource Center in the National Institute of Technology and Evaluation, Chiba, Japan. *A*. *baumannii* (JCM 6841) was purchased from RIKEN BioResource Research Center, Ibaraki, Japan. MRSA (IID 1677) was purchased from the Institute of Medical Science in the University of Tokyo, Japan. Lysozyme (Lysozyme BIO; purified from fresh chicken egg white) was purchased from Biocon Japan Ltd., Aichi, Japan. Chitosan oligosaccharide (COS) (made from shells of the crab) was purchased from Kimika Co., Aichi, Japan. COS presented a deacetylation degree of over 98% and a molecular weight (MW) of 5 kDa. Galactomannan was purchased from Taiyo Kagaku, Tokyo, Japan. Galactomannan presented an MW of 15 kDa. Precast sodium dodecyl sulfate (SDS) gel (SuperSep Ace 10–20%), the Wako Silver Stain II kit, colistin sulfate, vancomycin hydrochloride, N-phenyl-1-naphthylamine (NPN), 2-nitrophenyl β-D-galactopyranoside (ONPG), and *Micrococcus luteus* were purchased from FUJIFILM Wako Pure Chemical Corporation, Osaka, Japan. Defibrinated rabbit blood was purchased from Cosmo Bio Co., Tokyo, Japan. The LIVE/DEAD BacLight Bacterial Viability Kit (L7012) was purchased from Thermo Fisher Scientific Corporation, Massachusetts, USA.

### Production of lysozyme-chitosan oligosaccharide conjugate (LYZOX) and lysozyme-galactomannan conjugate

The lysozyme-COS powder mixtures were prepared by saturating the COS to maintain the mixing weight ratio of lysozyme at COS 1:1. The mixtures were dissolved in water at 100 mg/mL and freeze-dried. Powdered lysozyme-COS mixtures were dry-heated at 60°C under 65% relative humidity for fourteen days. The details were described previously [16]. LGC was produced using the same method. At 20±5°C, the saturated concentration of LYZOX in distilled water (DW) was 400 mg/mL, and the saturated concentration of LGC in DW was 300 mg/mL.

### Results of the Maillard reaction with lysozyme and chitosan oligosaccharide

Maillard reaction products (MRPs) have fluorescence properties. Their fluorescence intensity increases during the Maillard reaction [20]. The fluorescence intensity of LYZOX (500 μg/mL) and a mixture of lysozyme (250 μg/mL) and COS (250 μg/mL) were measured in triplicate at an excitation wavelength of 370 nm and an emission wavelength of 440 nm using a fluorescence spectrometer (RF-6000, Shimadzu, Kyoto, Japan). Then, SDS-polyacrylamide gel electrophoresis (SDS-PAGE) was carried out with a 10–20% gradient gel. LYZOX (10 μL, 500 μg/mL), lysozyme (10 μL, 250 μg/mL) and the mixture (10 μL, lysozyme [250 μg/mL] and COS [250 μg/mL]) were prepared in sample buffer (62.5 mM Tris-HCl, pH 6.8, 25% glycerol, 2% SDS, 10% 2-mercaptoethanol, and 0.02% bromophenol blue). Electrophoresis was performed at a current of 40 mA for 2 h in electrophoretic buffer containing 0.025 M Tris, 0.192 M glycine, and 0.1% SDS, pH 8.3. After electrophoresis, the gels were stained with a silver stain kit (Wako Silver Stain II kit) according to the manufacturer’s instructions.

### Antibacterial activity of LYZOX

#### Assays for bactericidal activity

LYZOX solution (2,000 μg/mL), the mixed solution (lysozyme solution [1,000 μg/mL] and COS solution [1,000 μg/mL]) and LGC solution (2,000 μg/mL) were prepared in saline. The pH of each solution was adjusted to a pH of 7.0–7.3 by adding 30 μL of 1.0 M phosphate buffer (pH 7.2) per 1.0 mL solution. Bacteria (*P*. *aeruginosa* [NBRC 13275 and PAO1], *A*. *baumannii* [JCM 6841] and MRSA [IID 1677]) were grown overnight at 37°C in tryptic soy broth (TSB) and collected in the exponential phase of growth by centrifugation. The bacteria were washed and resuspended. The final cell suspension was adjusted to an absorbance at 600 nm of 1.0 (10^8^−10^9^ CFU/mL). A volume of 0.1 mL of bacterial suspensions was incubated with each treatment solution in a volume of 10 mL at 37°C in a water bath for 0 min, 30 min, 60 min and 120 min. A volume of 0.1mL from each solution was plated after various incubation times and then the colonies were enumerated following growth overnight. Assays were performed as triplicate measurements from four independent experiments. As the weight ratio of lysozyme and COS in LYZOX was 1:1, the bactericidal activity of the LYZOX solution (2,000 μg/mL) was compared with that of the mixed solution (lysozyme solution [1,000 μg/mL] and COS solution [1,000 μg/mL]). To align the weight of lysozyme, the antibacterial activity of LGC was compared with the antibacterial activity of LYZOX at the same concentration because the weight ratio of lysozyme and galactomannan in LGC was also 1:1.

#### Growth inhibition test

LYZOX solution (2,000 μg/mL), lysozyme solution (1,000 μg/mL), COS solution (1,000 μg/mL), the mixed solution (lysozyme solution [1,000 μg/mL] and COS solution [1,000 μg/mL]) and LGC solution (2,000 μg/mL) were prepared in TSB [21]. The pH of each solution was adjusted to a pH of 6.8–7.3 by adding 10 μL of 1.0 M phosphate buffer (pH 7.2) per 1.0 mL solution. Bacteria (*P*. *aeruginosa* [NBRC 13275 and PAO1], *A*. *baumannii* and MRSA) were prepared as above. Bacteria were added to each treatment solution at 2.5×10^3^−10^4^ CFU in a volume of 10 mL and incubated aerobically at 37°C for 6 h [22]. The dilutions were plated after a 6-hour incubation, the colonies were counted following growth overnight, and the results were compared with the control. Assays were performed as duplicate measurements from 18 independent experiments for *P*. *aeruginosa* (NBRC 13275). Assays were also performed as duplicate measurements from eight independent experiments for *P*. *aeruginosa* (PAO1), *A*.*baumannii* and MRSA. We used a 6-hour incubation time because the variation of inhibition was large after 6 h (S1 Fig). To align the weight of lysozyme (or COS), we compared the antibacterial activity of lysozyme (or COS) with that of LYZOX at a concentration that was two-fold that of lysozyme (or COS).

### Mechanisms of action

#### Cell membrane integrity assays

The bacterial cell membrane integrity was examined by determining the release of material with absorption at 260 nm [23, 24]. Bacteria (*P*. *aeruginosa* [NBRC 13275], *A*. *baumannii* and MRSA) were prepared as described above, washed and resuspended in 0.5% NaCl solution. The final cell suspension was adjusted to an absorbance at 420 nm of 0.7. LYZOX solutions with different concentrations (4,000, 10,000 and 20,000 μg/mL) in 0.5% NaCl solution were prepared. The pH of each solution was adjusted to a pH of 6.6–7.1 by adding 60 μL of 1.0 M phosphate buffer (pH 7.2) per 1.0 mL solution. Ten milliliters of LYZOX solution at each concentration or 0.5% NaCl solution (control) was mixed with 10 mL of each bacterial cell suspension, and the mixed solutions were incubated at 37°C in a water bath. The mixed solutions were filtered with 0.22 μm syringe filters to remove the bacteria at various time points (20, 40, 60, 80, 100 and 120 min). The absorbances at 260 nm (*A*_260 nm_) of the supernatants were measured in triplicate.

#### NPN assays

The outer membrane permeability of the gram-negative bacteria (*P*. *aeruginosa* [NBRC 13275] and *A*. *baumannii*) and the plasma membrane permeability of MRSA were determined by NPN assays [25–27]. The bacteria were prepared as described above, washed and resuspended in 0.5% NaCl solution. The final cell suspension was adjusted to an absorbance at 420 nm of 1.0. LYZOX solutions with different concentrations (20 and 40 μg/mL) in 0.5% NaCl solution were prepared. The pH of each solution was adjusted to a pH of 7.2–7.3 by adding 60 μL of 1.0 M phosphate buffer (pH 7.2) per 1.0 mL solution. A 1.5 mL aliquot of LYZOX solution at each concentration or 0.5% NaCl solution (control) was mixed with 30 μL of 1.0 mM NPN solution. The fluorescence intensity at an excitation wavelength of 350 nm and an emission wavelength of 420 nm was measured with a fluorescence spectrophotometer (RF-6000, Shimadzu, Kyoto, Japan) for 10 min. Three independent experiments were performed for each type of bacteria in control solution and LYZOX solution at each concentration. Low concentrations of LYZOX were used to reduce the fluorescence of LYZOX itself to measure the fluorescence intensity of NPN. The fluorescence intensity of LYZOX solution at each concentration without bacterial suspension (1.5 mL of LYZOX solution, 30 μL of NPN and 1.5 mL of 0.5% NaCl) was subtracted from the fluorescence intensity of the corresponding LYZOX solution with bacterial suspension (1.5 mL of LYZOX solution, 30 μL of NPN and 1.5 mL of bacterial suspension) to determine the fluorescence intensity emitted by NPN. The calculated results were expressed as relative fluorescence units.

#### ONPG assays

The inner membrane permeabilization of the gram-negative bacteria (*P*. *aeruginosa* [NBRC13275] and *A*. *baumannii*) and the plasma membrane permeabilization of MRSA were determined by measuring the activity of cytoplasmic β-galactosidase released from bacteria into the LYZOX solutions using ONPG as a substrate [23]. The bacteria were prepared as described above, washed and resuspended in 0.5% NaCl solution. The final cell suspension was adjusted to an absorbance at 420 nm of 1.2. LYZOX solutions with different concentrations (400, 4,000 and 10,000 μg/mL) in 0.5% NaCl solution were prepared. The pH of each solution was adjusted to a pH of 6.8–7.3 by adding 60 μL of 1.0 M phosphate buffer (pH 7.2) per 1.0 mL solution. A 1.6 mL aliquot of LYZOX solution at each concentration or 0.5% NaCl solution (control) was mixed with 1.6 mL of each bacterial cell suspension and 150 μL of 30 mM ONPG solution. The production of o-nitrophenol over time was determined by monitoring the increase in absorption at 420 nm (*A*_420 nm_) in triplicate.

#### LIVE/DEAD bacterial viability assays

Confocal laser scanning microscopy (CLSM) was used to visualize the damaged membranes of bacteria after LYZOX treatment [28]. LYZOX solution (2,000 μg/mL) in saline was prepared and adjusted to pH 7.0–7.1 by adding 60 μL of 1.0 M phosphate buffer (pH 7.2) per 1.0 mL solution. The bacterial cell suspensions (*P*. *aeruginosa* [NBRC13275], *A*. *baumannii*, and MRSA) were adjusted to an absorbance at 600 nm of 1.0. A 500 μL aliquot of LYZOX solution or saline (control) was mixed with 500 μL of each bacterial cell suspension, and the mixed solutions were incubated at 37°C for 2 h. After incubation, 3 μL of LIVE/DEAD BacLight reagent was added to 1.0 mL of incubated solution and further incubated at room temperature for 15 min. The LIVE/DEAD BacLight Bacterial Viability kit utilized mixtures of SYTO 9 and propidium iodide (PI) to stain nucleic acids. Green-fluorescing SYTO 9 generally labels all bacteria, while PI penetrates only dead or dying bacteria with damaged cytoplasmic membranes, causing a reduction in the fluorescence of SYTO 9 when both dyes are present. The bacterial cells were imaged using a Leica TCS SP8 CLSM (Leica Microsystems, Wetzlar, Germany) with a 63× oil immersion lens. The excitation/emission wavelengths were 488/495-515 nm for SYTO 9 and 488/635-700 nm for PI.

#### Electron microscopy

The effects of LYZOX on the morphology of *P*. *aeruginosa* (NBRC 13275), *A*. *baumannii* and MRSA cells were evaluated by scanning electron microscopy (SEM) and transmission electron microscopy (TEM). For SEM, each bacterial suspension was adjusted to an absorbance of 2.0 at 600 nm. For TEM, each bacterial suspension was prepared by adjusting a 10-fold dilution to an absorbance of 0.4 at 600 nm. The bacterial cells were treated with LYZOX (1,000 μg/mL), lysozyme (500 μg/mL), COS (500 μg/mL) or a mixture (lysozyme [500 μg/mL] and COS [500 μg/mL]). A negative control (absence of treatment) is shown for comparison. Bacteria were incubated with each treatment for 2 h at 37°C. After centrifugation, the pellets were rinsed with saline and fixed with fixative solution (2.5% buffered glutaraldehyde in 0.1 M phosphate buffer) for 2 h at 4°C. Then, samples were rinsed and incubated in 0.1 M phosphate buffer overnight. For SEM, the bacteria cells were postfixed in 1.0% buffered osmium tetroxide in 0.1 M phosphate buffer for 1 h and then dehydrated in a sequential ethanol series (50, 70, 80, 90, 95 and 100%; each percentage except 100% was applied for 10 min, and 100% was applied three times for 10 min each time) and dried in a critical point drying apparatus (HCP-2; Hitachi, Tokyo, Japan) with liquid CO_2_. After vacuum sputter-coating with approximately 20 nm of platinum/palladium, the samples were observed by scanning electron microscope (S-4500 Hitachi, Tokyo, Japan). For TEM, the bacteria were fixed in 1.0% buffered osmium tetroxide in 0.1 M phosphate buffer for 1 h, embedded in 2.0% agar, dehydrated in a sequential ethanol series as described above and then embedded in Epon 812 (TAAB Laboratories Equipment Ltd., Berkshire, England). Ultrathin sections of 70 nm were collected on copper grids, double-stained with uranyl acetate and lead citrate and observed by transmission electron microscope (H-7100 Hitachi, Tokyo, Japan) at 75 kV.

#### Magnesium effect

To confirm a chelating agent of LYZOX, magnesium assays were performed as described previously [29]. LYZOX solution (10,000 μg/mL) and COS solution (10,000 μg/mL) were prepared in TSB and adjusted to 0.05 M magnesium chloride solution. As the control, TSB without treatment was prepared and adjusted to 0.05 M magnesium chloride solution. The pH of each solution was adjusted to a pH of 6.4–6.9 by adding 10 μL of 1.0 M phosphate buffer (pH 7.2) per 1.0 mL solution. Bacteria (*P*. *aeruginosa* [NBRC 13275] and MRSA) were prepared as described above, washed and resuspended in saline. The final cell suspension was adjusted to an absorbance at 600 nm of 1.0. A volume of 0.1 mL of bacterial suspension was incubated with each treatment solution in a volume of 5.0 mL at 37°C for 10 h. Each solution was then measured spectrometrically at 600 nm (*A*_600 nm_) and compared with the value of the control. Three independent experiments were performed for *P*. *aeruginosa* (NBRC 13275) and MRSA. To evaluate the effect of magnesium, we performed the same experiments without magnesium.

### Comparison of the minimal inhibitory concentrations

*P*. *aeruginosa* (NBRC 13275 and PAO1), *A*. *baumannii* and MRSA were cultured in LB broth containing LYZOX, and the minimal inhibitory concentration (MIC) of LYZOX was evaluated using a modified method based on that described by CLSI (Clinical and Laboratory Standards Institute) [15, 30]. A total of 60 μL of each bacterial suspension in PBS was added to 140 μL of LB broth containing LYZOX. Bacteria at a final concentration of 10^4^ CFU/mL in diluted LB broth with different concentrations of LYZOX were incubated at 35°C for 18 h. The MIC was defined as the lowest drug concentration that allowed no visual growth of bacteria. The MICs of the mixed solution (lysozyme and COS) and the conventional antibiotics were also evaluated for comparison with that of LYZOX as described above. The conventional antibiotics we used were vancomycin, which is one of the therapeutic agents used in the treatment of MRSA infections, and colistin, which is one of the therapeutic agents used to treat both multidrug-resistant (MDR) *P*. *aeruginosa* and MDR *Acinetobacter* infections [31].

### Acquired drug resistance test

To evaluate the development of drug resistance, *P*. *aeruginosa* (NBRC 13275 and PAO1), *A*. *baumannii* and MRSA were subcultured repeatedly in LB broth containing LYZOX, and the change of susceptibility to LYZOX was evaluated [32]. The MIC of LYZOX was measured as described above. We repeatedly grew bacteria in a half of MIC for LYZOX, isolated the bacteria, and subcultured it, and each isolated bacterium was evaluated for its MIC for LYZOX. Each bacterium not subcultured was also evaluated as the control. The measurement of MIC was repeated 10 times and we evaluated the development of drug resistance in these bacteria. The mixture (lysozyme and COS) was also evaluated for comparison with LYZOX as described above.

### Hemolytic toxicity test

Defibrinated blood from rabbits was diluted by mixing 400 μL of blood with 10 mL of PBS. Each concentration of LYZOX solution (20, 200, 2,000 and 20,000 μg/mL) was prepared in PBS. Five hundred microliters of diluted blood was mixed with 500 μL of each LYZOX solution, PBS (negative control) and 0.1% Triton-X 100 in PBS (positive control). The samples were incubated for 1 h at 37°C followed by centrifugation for 10 min at 1500 rpm, and the supernatants were collected. Then, 750 μL of each sample was added to 750 μL of PBS, and 750 μL of control (positive or negative control) was added to 750 μL of each corresponding concentration of LYZOX because the absorbances of LYZOX at 545 nm were different at each concentration. Each OD was measured at 545 nm to calculate the hemolysis rate by using the following equation: Hemolytic rate (HR) = (Absorbance of sample [AS]–Absorbance of negative control with the corresponding concentration of LYZOX [AN]) / (Absorbance of positive control with the corresponding concentration of LYZOX [AP]–AN) [28, 33]. AS, AN, and AP are the OD values of the adjusted supernatants from the test samples, the negative control and the positive control, respectively.

### Heat stability of lysozyme and LYZOX

The effect of heat on the antibacterial activity of lysozyme and LYZOX was evaluated by measuring the lytic activity against *Micrococcus luteus* in a turbidimetric analysis [34, 35]. Lysozyme hydrolyses peptidoglycan which is one of the bacterial wall components [8]. First, lysozyme and LYZOX were exposed to 80°C for various times (60, 120, 180 and 240 min). The lytic activity of heated agents (1,000 μg/mL) for 10 min was evaluated by measuring the turbidity at optical density (OD) at 600 nm. Second, we examined the lytic activity of lysozyme and LYZOX with or without heat treatment at 80°C for 120 min (1,000 μg/mL, respectively).

### Statistical analysis

Statistical analyses were performed using GraphPad Prism 7 software (GraphPad, California, USA). Experimental values were expressed as the mean and standard error of the mean (SEM). Student’s t-tests were used for comparisons between the two groups. A p value less than 0.05 was regarded as statistically significant.

## Results

### Results of the Maillard reaction with lysozyme and chitosan oligosaccharide

The formation of LYZOX via the Maillard reaction was confirmed by measuring the fluorescence intensity and performing SDS-PAGE. When lysozyme was conjugated to COS via the Maillard reaction, the fluorescence intensity was significantly increased from 11.43±0.12 to 277.8±1.52 (p<0.01) (Fig a in S2 Fig). SDS-PAGE analysis of lysozyme revealed a major band at 14 kDa in lane 1 (Fig b in S2 Fig). Weak bands at low molecular weights and at 18 kDa were also observed in lane 1. Identical bands appeared in the lane containing the mixture (lane 2) that were of the same molecular weight as lysozyme. At 28 kDa and the similar molecular weights as lysozyme and mixture (14 kDa and low molecular weights), identical but broader bands appeared in the lane containing LYZOX (lane 3). Furthermore, extensive, diffuse bands were also observed that ranged from low molecular weights to over 45 kDa for LYZOX (lane 3). It was confirmed that the fluorescence intensity and the molecular weight were increased after lysozyme was conjugated to COS via the Maillard reaction.

### Antibacterial activity of LYZOX

#### Assays for bactericidal activity

**Fig 1:**
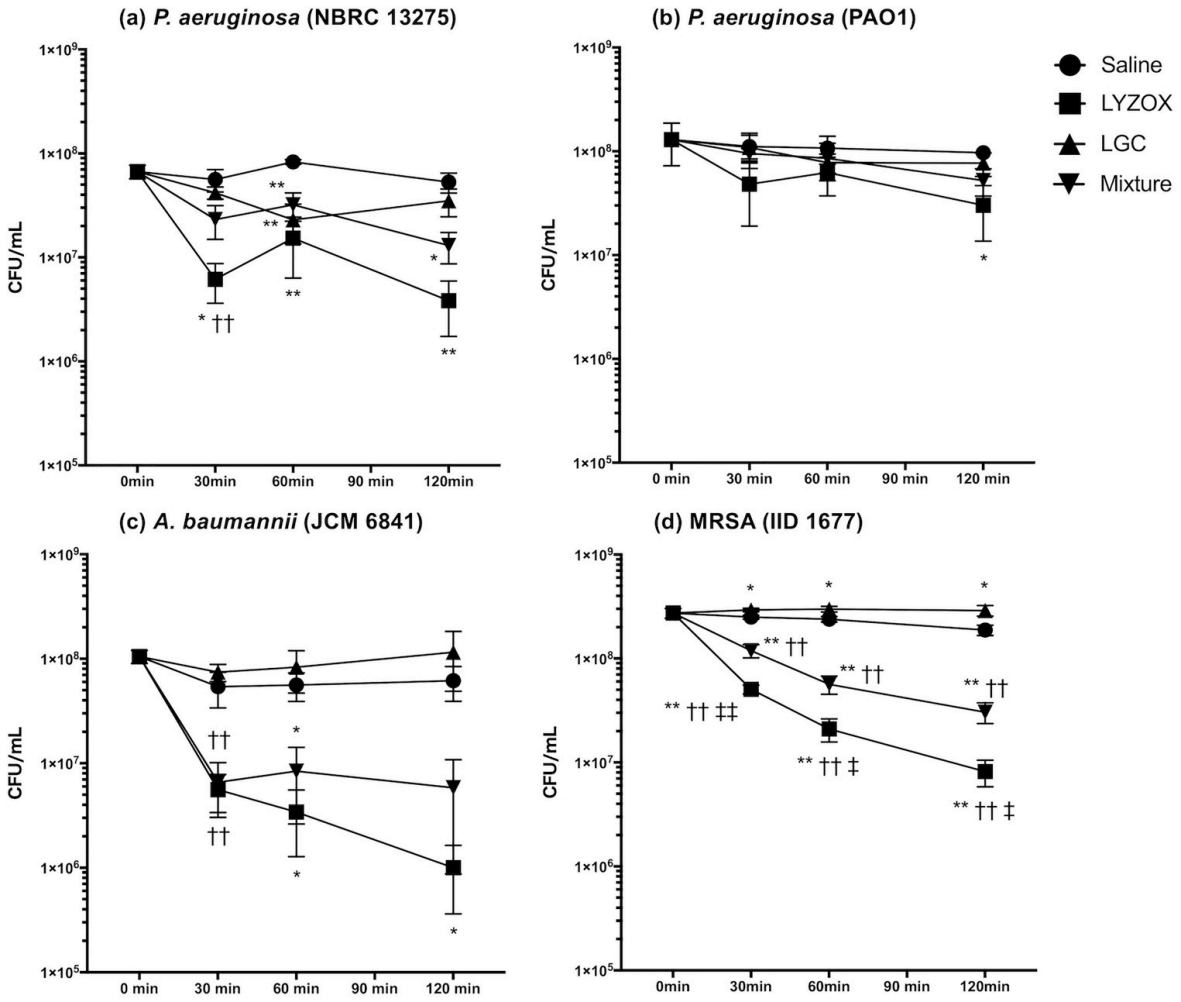
Assays for bactericidal activity. (a) *Pseudomonas aeruginosa* (NBRC 13275), (b) *Pseudomonas aeruginosa* (PAO1), (c) *Acinetobacter baumannii* (JCM 6841), (d) MRSA (IID 1677). Each type of bacteria was incubated with LYZOX solution (2,000 μg/mL), the mixed solution (lysozyme solution [1,000 μg/mL] and chitosan oligosaccharide [COS] solution [1,000 μg/mL]) or the lysozyme-galactomannan conjugate (LGC) solution (2,000 μg/mL) in saline at 37°C in a water bath for 0 min, 30 min, 60 min and 120 min. The dilutions were plated, and the colonies were counted following growth overnight. The values are the mean ± SEM from four independent experiments. *p<0.05 or **p<0.01 compared with saline; †p<0.05 or ††p<0.01 compared with LGC; ‡p<0.05 or ‡‡p<0.01 compared with the mixture (unpaired t-test). Symbols: circles, saline; squares, lysozyme-chitosan oligosaccharide conjugate (LYZOX); up-pointing triangles, LGC; down-pointing triangles, mixture.

We performed assays for bactericidal activity to evaluate bactericidal activities of LYZOX, a mixture (lysozyme and COS), and LGC against *P*. *aeruginosa* (NBRC 13275 and PAO1), *A*. *baumannii* (JCM 6841), and MRSA (IID 1677) (Fig 1). LYZOX was significantly more effective than the control against *P*. *aeruginosa* (NBRC 13275) at 30 min, 60 min, and 120 min (LYZOX, p = 0.014, p = 0.001, and p = 0.006, respectively). LYZOX was significantly more effective than the control against *P*. *aeruginosa* (PAO1) at 120 min (p = 0.024). LYZOX was significantly more effective than the control against *A*. *baumannii* at 60 min and 120 min (p = 0.022 and p = 0.036, respectively). LYZOX and the mixture were significantly more effective than the control against MRSA at 30 min, 60 min, and 120 min (LYZOX, p<0.001, p<0.001, and p<0.001; mixture, p = 0.001, p = 0.028, and p = 0.022, respectively). LYZOX was significantly more effective than the mixture against MRSA at 30 min, 60 min and 120 min (p = 0.009, p = 0.028, p = 0.022, respectively). There were no significant differences in the bactericidal activity between LYZOX and the mixture against *P*. *aeruginosa* (NBRC 13275 and PAO1) and *A*. *baumannii*. LYZOX killed *P*. *aeruginosa* (NBRC 13275 and PAO1), *A*. *baumannii*, and MRSA 50% more than the control at 30 min, 120 min, 60 min and 30 min. The mixture was significantly more effective than the control against *A*. *baumannii* at 60 min (p = 0.039). Bacterial counts in MRSA treated with LGC were significantly increased compared with the control at 30 min, 60 min, and 120 min. (p = 0.029, p = 0.045, and p = 0.044, respectively). LGC was not confirmed to possess effective antibacterial activity compared with the other treatments. LYZOX demonstrated concentration-dependent bactericidal activity (S3 Fig).

#### Growth inhibition test

**Fig 2:**
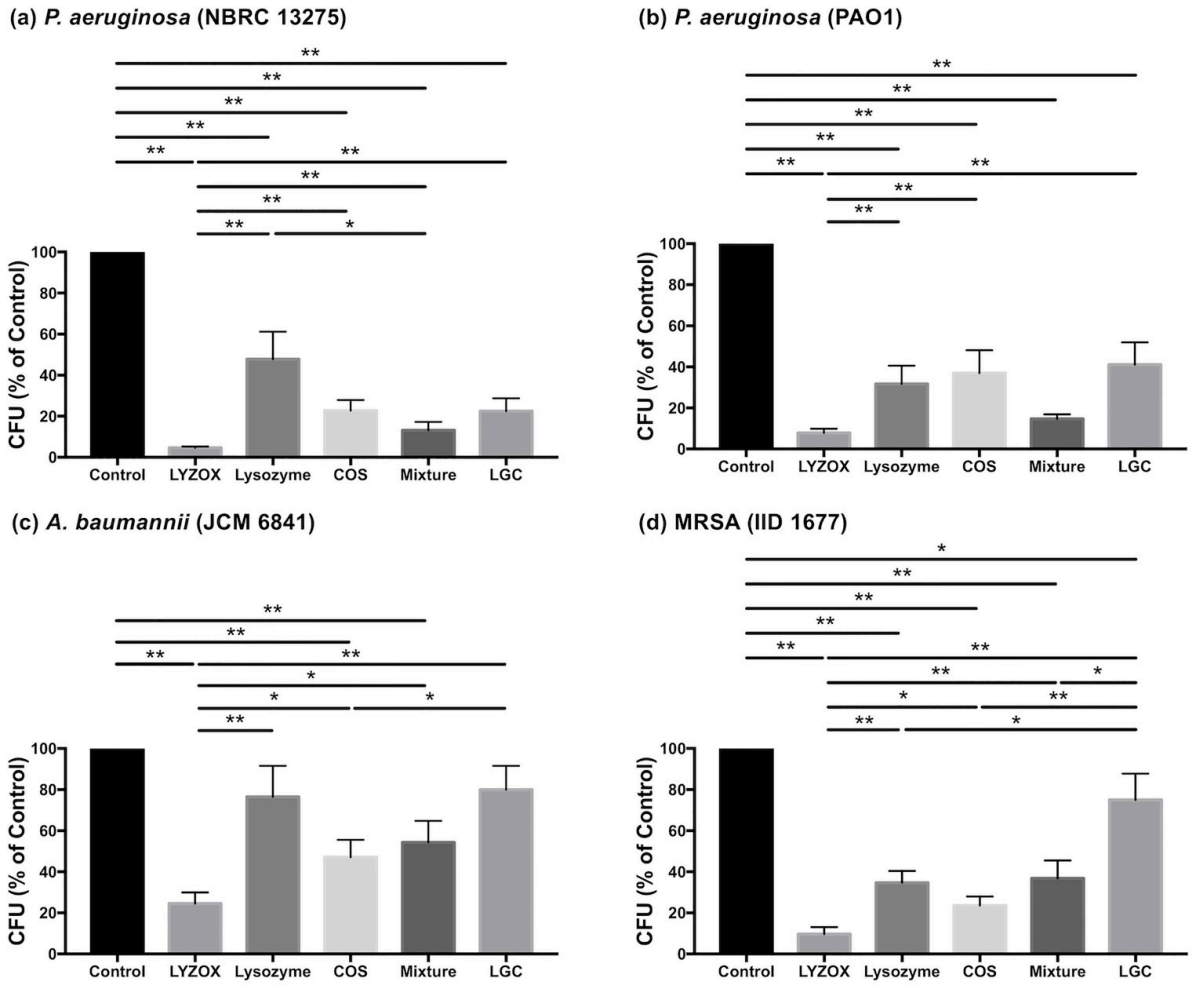
Growth inhibition test. (a) *Pseudomonas aeruginosa* (NBRC 13275), (b) *Pseudomonas aeruginosa* (PAO1), (c) *Acinetobacter baumannii* (JCM 6841), (d) MRSA (IID 1677). Each type of bacteria was incubated with LYZOX solution (2,000 μg/mL), lysozyme solution (1,000 μg/mL), chitosan oligosaccharide (COS) solution (1,000 μg/mL), the mixed solution (lysozyme solution [1,000 μg/mL] and COS solution [1,000 μg/mL]) or lysozyme-galactomannan conjugate solution (2,000 μg/mL) in tryptic soy broth (TSB) at 37 °C for 6 h. The dilutions were plated, the colonies were counted following growth overnight, and results were compared with the control. The control was TSB without each treatment. The values are the mean ± SEM from 18 independent experiments for *P*. *aeruginosa* (NBRC 13275). The values are the mean ± SEM from eight independent experiments for *P*. *aeruginosa* (PAO1), *A*. *baumannii* and MRSA. *p<0.05, **p<0.01 (unpaired t-test). LYZOX: lysozyme-chitosan oligosaccharide conjugate. LGC: lysozyme-galactomannan conjugate.

We tested the activity of LYZOX, lysozyme, COS, the mixture, and LGC against *P*. *aeruginosa* (NBRC 13275 and PAO1), *A*. *baumannii*, and MRSA (Fig 2). The control contained TSB without any treatment. Each treatment, except lysozyme and LGC in *A*. *baumannii*, was significantly more effective than the control in inhibiting the growth of each type of bacteria (in *P*. *aeruginosa* [NBRC 13275]: LYZOX, p<0.001; lysozyme, p<0.001; COS, p<0.001; mixture, p<0.001; LGC, p<0.001; in *P*. *aeruginosa* [PAO1]: LYZOX, p<0.001; lysozyme, p<0.001; COS, p<0.001; mixture, p<0.001; LGC, p<0.001; in *A*. *baumannii*: LYZOX, p<0.001; COS, p<0.001; mixture, p<0.001; in MRSA: LYZOX, p<0.001; lysozyme, p<0.001; COS, p<0.001; mixture, p<0.001; LGC, p = 0.015; all compared with the control). LYZOX was significantly more effective than the other treatments, except the mixture in *P*. *aeruginosa* (PAO1) (In *P*. *aeruginosa* [NBRC 13275]: lysozyme, p<0.001; COS, p<0.001; mixture, p = 0.003; LGC, p<0.001; in *P*. *aeruginosa* [PAO1]: lysozyme, p = 0.005; COS, p = 0.005; LGC, p = 0.002; in *A*. *baumannii*: lysozyme, p = 0.006; COS, p = 0.04; mixture, p = 0.024; in MRSA: lysozyme, p = 0.002; COS, p = 0.034; mixture, p = 0.005; LGC, p<0.001, all compared with LYZOX). LGC was significantly less effective than the other treatments in MRSA (LYZOX, p<0.001; lysozyme, p = 0.028; COS, p = 0.009; mixture, p = 0.048: all compared with LGC). Lysozyme was significantly less effective than the mixture in *P*. *aeruginosa* (NBRC 13275) (p = 0.038). COS was significantly more effective than LGC in *A*. *baumannii* (p = 0.038). LYZOX demonstrated concentration-dependent growth inhibition (S1 Fig).

### Mechanisms of action

#### Cell membrane integrity assays

**Fig 3:**
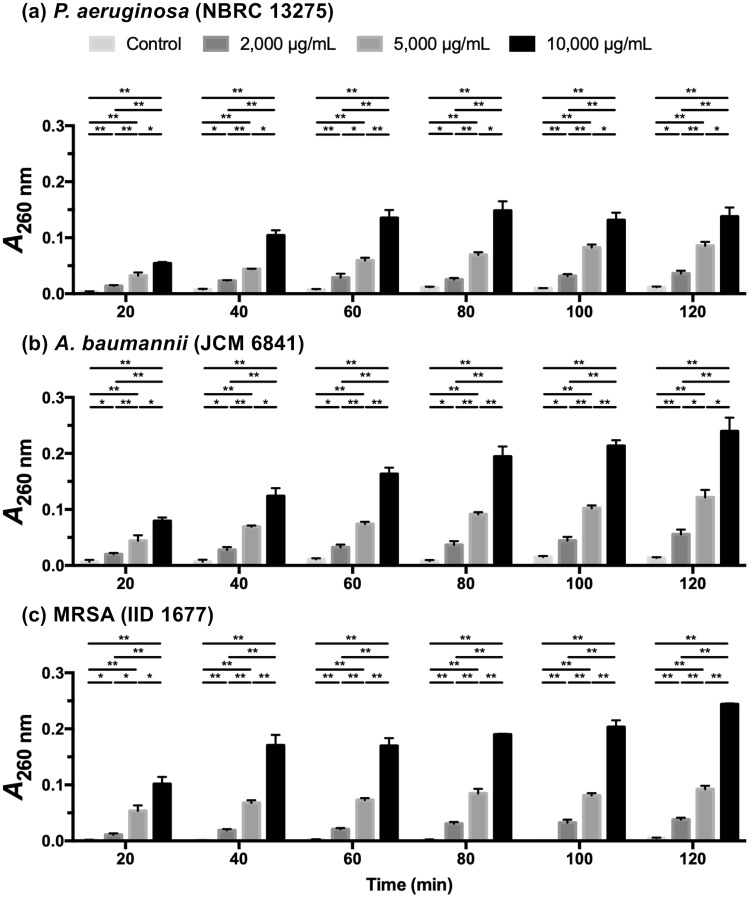
Cell membrane integrity assays. The measurement of the absorbance at 260 nm (*A*_260 nm_) can be used to estimate the amount of nucleic acids released from the cytoplasm, which can be used to evaluate the cell membrane integrity. (a) *Pseudomonas aeruginosa* (NBRC 13275), (b) *Acinetobacter baumannii* (JCM 6841), (c) MRSA (IID 1677). The values are the mean ± SEM of triplicate measurements. *p<0.05, **p<0.01 (unpaired t-test).

Intracellular components will be released into the extracellular space when the bacterial cell membrane is compromised by antibacterial agents. The measurement of absorbance at 260 nm can be used to estimate the amount of DNA and RNA released from the cytoplasm [23, 24]. The *A*_260 nm_ was shown to increase in a dose-dependent manner (Fig 3). When each bacterial suspension was treated with LYZOX (2,000, 5,000 and 10,000 μg/mL), the *A*_260 nm_ increased rapidly for the first 20 min then at a decreasing rate up to 120 min. When *P*. *aeruginosa* was treated with 10,000 μg/mL of LYZOX, there was no further increase in the *A*_260 nm_ after 80 min. The leakage of nucleic acids indicated that the bacterial membranes became compromised within 20 min of contact with LYZOX.

#### NPN assays

**Fig 4:**
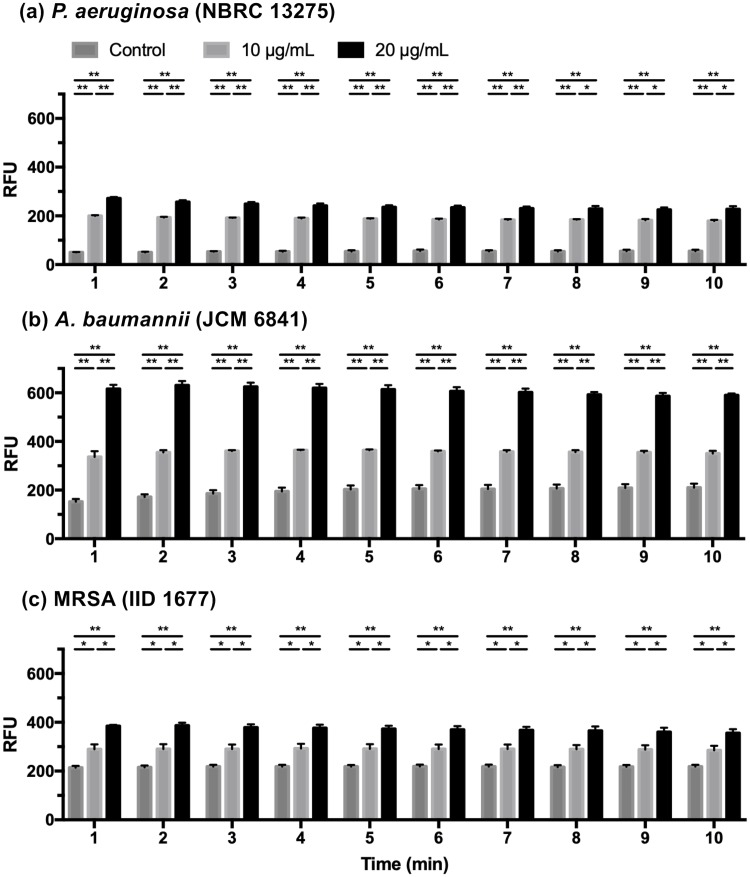
NPN assays. The outer membrane permeability of the gram-negative bacteria and plasma membrane permeability of MRSA were determined by NPN assays. (a) *Pseudomonas aeruginosa* (NBRC 13275), (b) *Acinetobacter baumannii* (JCM 6841), (c) MRSA (IID 1677). The values are the mean ± SEM. Three independent experiments were performed for each type of bacteria in the control solution and at each concentration of LYZOX. *p<0.05, **p<0.01 (unpaired t-test). RFU: relative fluorescence units.

NPN fluoresces strongly in phospholipid environments but only weakly in aqueous environments. This property is utilized to evaluate the permeability of the outer membranes of gram-negative bacteria [23, 25, 27]. Some previous studies have performed NPN assays to evaluate membrane damage in gram-positive bacteria [26, 36]. The results of the NPN assays are shown in Fig 4. In each type of bacteria, the fluorescence intensity increased after 1 min and remained at the same level thereafter. The addition of LYZOX to each type of bacteria in the presence of NPN caused a dose-dependent increase in fluorescence. These results showed that LYZOX increased the outer membrane permeability of gram-negative bacteria and the plasma membrane permeability of MRSA within one minute of contact with the bacteria.

#### ONPG assays

**Fig 5:**
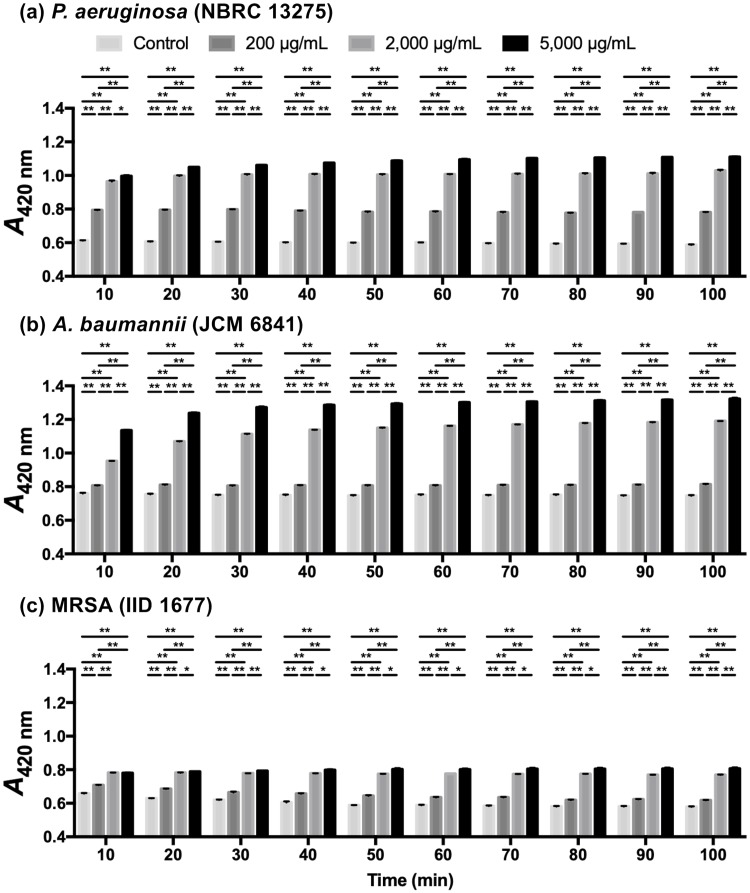
ONPG assays. The measurement of absorbance at 420 nm (*A*_420 nm_) can be used to estimate the activity of cytoplasmic β-galactosidase released from bacteria into LYZOX solution using ONPG as the substrate to evaluate inner membrane permeabilization in gram-negative bacteria and plasma membrane permeabilization in gram-positive bacteria. (a) *Pseudomonas aeruginosa* (NBRC 13275), (b) *Acinetobacter baumannii* (JCM 6841), (c) MRSA (IID 1677). The values are the mean ± SEM of triplicate measurements. *p<0.05, **p<0.01 (unpaired t-test).

Intracellular enzymes, including β-galactosidase, leak outside of the cell when the cell membrane is damaged. ONPG is normally colorless, but it is hydrolyzed by β-galactosidase to galactose and o-nitrophenol, which can be measured by determining the absorbance at 420 nm. In previous studies, the inner membrane permeabilization of gram-negative bacteria was determined by measuring the activity of cytoplasmic β-galactosidase released from bacteria using ONPG as the substrate [23]. Some previous studies have used ONPG assays to evaluate plasma membrane damage in gram-positive bacteria [26, 37]. The results of the ONPG assays are shown in Fig 5. When bacterial suspensions of *P*. *aeruginosa* and *A*. *baumannii* were treated with LYZOX (2,000 and 5,000 μg/mL), the *A*_420 nm_ increased rapidly at first and then at a decreasing rate up to 100 min. In *P*. *aeruginosa* and *A*. *baumannii* treated with LYZOX (200 μg/mL), the *A*_420 nm_ increased during the first 10 min and remained constant thereafter. In MRSA treated with LYZOX, there was no further increase in the *A*_420 nm_ after 10 min. In each type of bacteria, the *A*_420 nm_ increased in a dose-dependent manner. Intracellular enzymes leaked into the extracellular space in a dose-dependent manner within ten minutes of contact, indicating that LYZOX damaged the inner membrane of gram-negative bacteria and the plasma membrane of MRSA.

#### LIVE/DEAD bacterial viability assays

**Fig 6:**
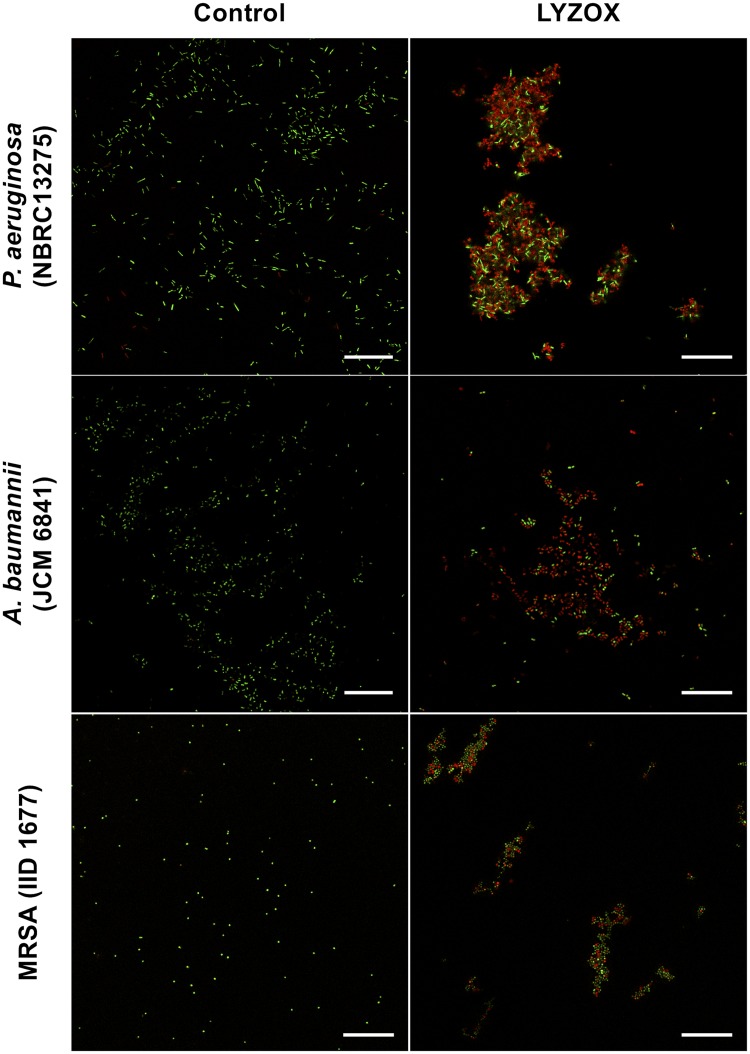
LIVE/DEAD bacterial viability assays. (a) *Pseudomonas aeruginosa* (NBRC 13275), (b) *Acinetobacter baumannii* (JCM 6841), (c) MRSA (IID 1677). Each type of bacteria was incubated in lysozyme-chitosan oligosaccharide conjugate (LYZOX) solution (1,000 μg/mL) or saline at 37°C for 2 h. Bacteria with intact cell membranes were stained with green fluorescent SYTO 9, whereas bacteria with damaged membranes were stained with red fluorescent propidium iodide (PI). The excitation/emission wavelengths were 488/495-515 nm for SYTO 9 and 488/635-700 nm for PI. Scale bar = 25 μm.

To confirm the membrane damage results described above, bacteria treated with LYZOX were visualized using CLSM (Fig 6). In each control sample, almost all bacteria were intact (green-fluorescent), although a few bacteria had damaged membranes (red-fluorescent). In each sample treated with LYZOX, there were more bacterial cells with damaged membranes than in the corresponding control sample. In *P*. *aeruginosa* and MRSA treated with LYZOX, intact bacterial cells and bacterial cells with damaged membranes aggregated and formed clusters. In contrast, in *A*. *baumannii* treated with LYZOX, there were fewer bacterial cell clusters compared to *P*. *aeruginosa* and MRSA. CLSM could visualize membrane damage due to LYZOX that was consistent with the results of the membrane damage assay described above.

#### Electron microscopy

To visualize the morphological changes in each type of bacteria treated with LYZOX, we examined bacteria treated with each solution using SEM and TEM.

#### Electron microscopic findings in *Pseudomonas aeruginosa* and *Acinetobacter baumannii*

**Fig 7:**
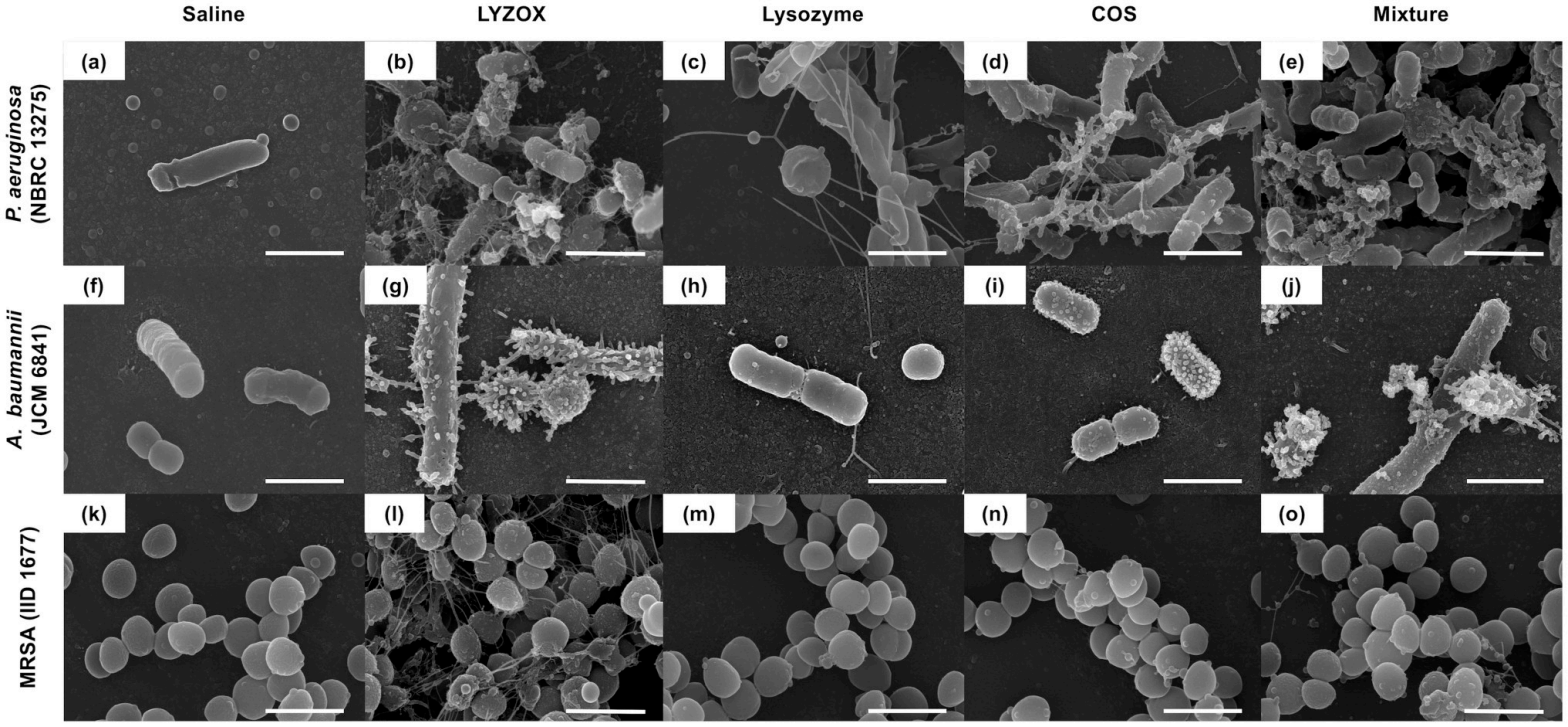
Scanning electron microscopic findings in *Pseudomonas aeruginosa*, *Acinetobacter baumannii* and methicillin-resistant *Staphylococcus aureus*. Scanning electron micrographs of *Pseudomonas aeruginosa* (NBRC 13275) (a, b, c, d, e), *Acinetobacter baumannii* (JCM 6841) (f, g, h, i, j) and MRSA (IID 1677) (k, l, m, n, o) incubated with each treatment for 2 h. (a), (f) and (k) were not treated (control). (b), (g) and (l) were treated with lysozyme-chitosan oligosaccharide conjugate (LYZOX, 1,000 μg/mL). (c), (h) and (m) were treated with lysozyme (500 μg/mL). (d), (i) and (n) were treated with chitosan oligosaccharide (COS, 500 μg/mL). (e), (j) and (o) were treated with the mixture (lysozyme [500 μg/mL] and COS [500 μg/mL]). Bacteria were photographed at a magnification of ×20,000. Scale bar = 1.5 μm.

**Fig 8:**
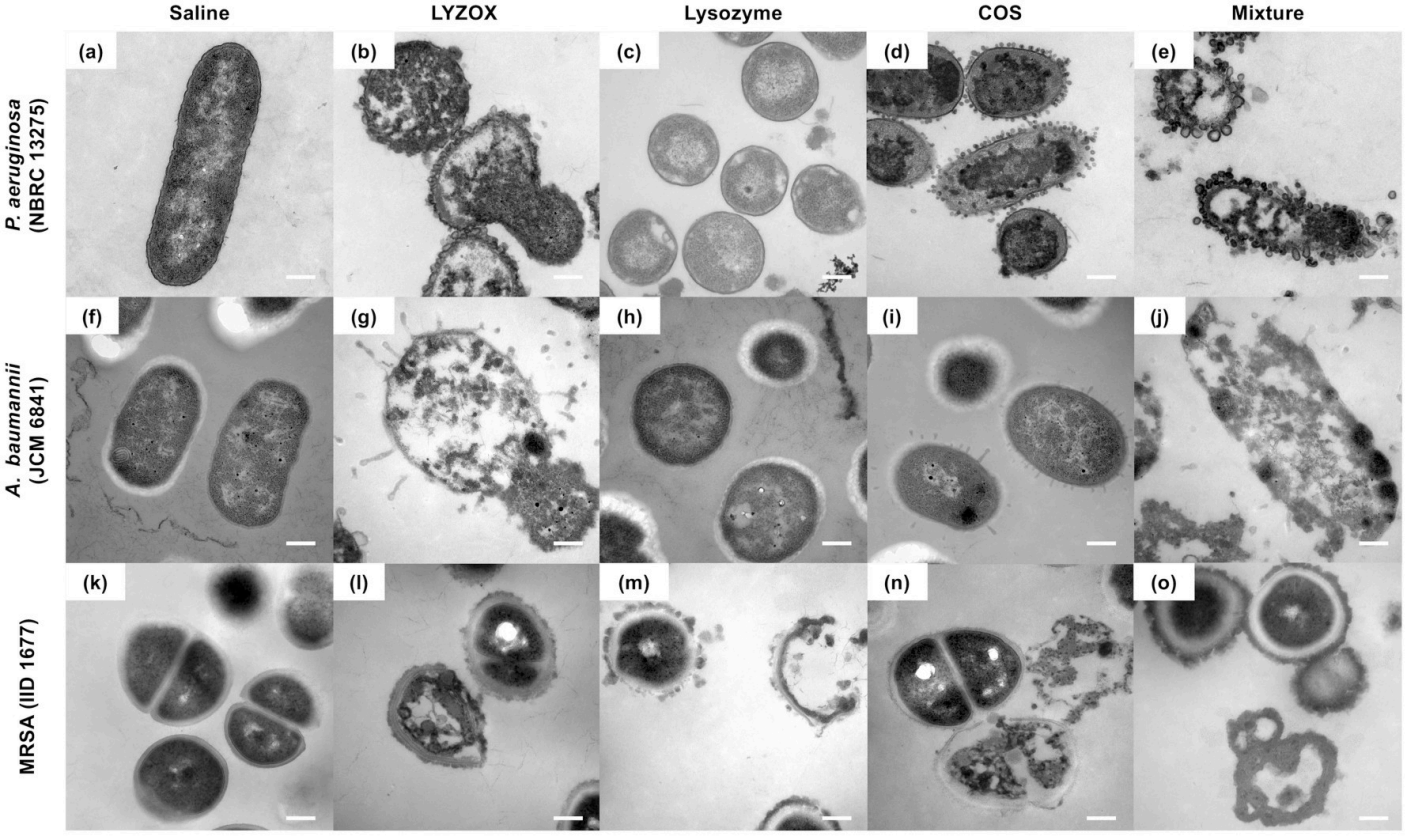
Transmission electron microscopic findings in *Pseudomonas aeruginosa*, *Acinetobacter baumannii* and methicillin-resistant *Staphylococcus aureus*. Transmission electron micrographs of *Pseudomonas aeruginosa* (NBRC 13275) (a, b, c, d, e), *Acinetobacter baumannii* (JCM 6841) (f, g, h, i, j) and MRSA (IID 1677) (k, l, m, n, o) incubated with each treatment for 2 h. (a), (f) and (k) were not treated (control). (b), (g) and (l) were treated with lysozyme-chitosan oligosaccharide conjugate (LYZOX, 1,000 μg/mL). (c), (h) and (m) were treated with lysozyme (500 μg/mL). (d), (i) and (n) were treated with chitosan oligosaccharide (COS, 500 μg/mL). (e), (j) and (o) were treated with the mixture (lysozyme [500 μg/mL] and COS [500 μg/mL]). Bacteria were photographed at a magnification of of ×50,000. Scale bar = 0.2 μm.

SEM micrographs of untreated *P*. *aeruginosa* (NBRC 13275) and *A*. *baumannii* displayed a smooth surface (Fig 7a and 7f). After treatment with lysozyme, *P*. *aeruginosa* and *A*. *baumannii* partly formed spheroplasts, which were round cells (Fig 7c and 7h). Many blebs were observed for both *P*. *aeruginosa* and *A*. *baumannii* treated with LYZOX (Fig 7b and 7g) compared to cells treated with COS and the mixture (Fig 7d, 7e, 7i and 7j). *P*. *aeruginosa* and *A*. *baumannii* treated with LYZOX and the mixture lost the integrity of their surface structure and became partially spherical (Fig 7b, 7e, 7g and 7j). After treatment with each solution, particularly treatment with LYZOX, extracellular thread-like structures appeared around the cells (Fig 7b–7e and 7g–7j). TEM micrographs of untreated *P*. *aeruginosa* and *A*. *baumannii* illustrated the normal surface architecture of the bacterium, which appears smooth and sharply layered (Fig 8a and 7f). Treatment with lysozyme caused both *P*. *aeruginosa* and *A*. *baumannii* to form spheroplasts (Fig 8c and 8h), and treatment with COS caused these bacteria form many vesicles on their surfaces (Fig 8d and 8i). As vesicles are generated from outer membranes, these corresponded to the blebs observed in the SEM micrographs of each bacterium. After treatment with LYZOX or the mixture, TEM micrographs of *P*. *aeruginosa* and *A*. *baumannii* revealed the morphological changes that resulted from both lysozyme and COS treatment by showing spherical cells with outer membrane vesicles (Fig 8b, 8e, 8g and 8j). Additionally, bacterial cell walls were destroyed and intracellular contents were released from the cells.

#### Electron microscopic findings in methicillin-resistant *Staphylococcus aureus*

SEM micrographs of untreated MRSA illustrated the normal surface architecture of the bacterium (Fig 7k). MRSA mostly maintained its surface structure in each treatment solution (Fig 7l–7o). After treatment with each solution, with the exception of lysozyme, extracellular thread-like structures appeared around the cells that were similar to those seen in gram-negative bacteria (Fig 7l, 7n and 7o); this was particularly true in the case of LYZOX. TEM micrographs of untreated MRSA illustrated the normal surface architecture of the bacterium, which appears smooth and sharply layered (Fig 8k). Each treatment affected the bacterial cell walls, which were thickened compared to those of the control (Fig 8l–8o). Each treatment destroyed the bacterial cell walls and led to the loss of cellular contents (Fig 8l–8o).

#### Magnesium effect

**Fig 9:**
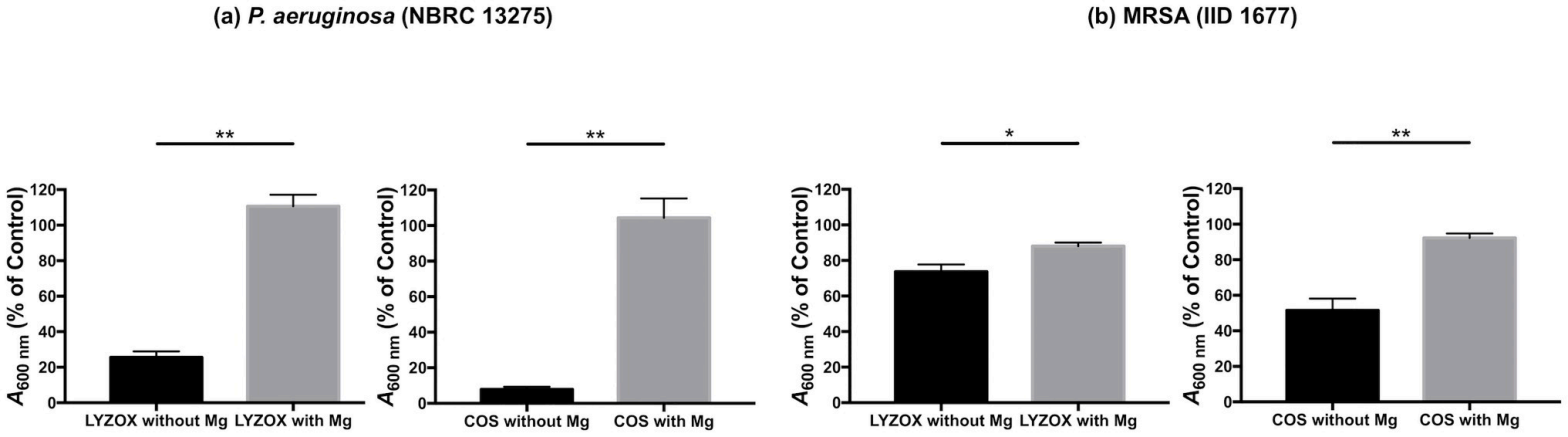
Magnesium effect. (a) *Pseudomonas aeruginosa* (NBRC 13275), (b) MRSA (IID 1677). Each type of bacteria was incubated with each treatment solution (LYZOX [10,000 μg/mL] and COS [10,000 μg/mL]) and magnesium (0.05 M) in tryptic soy broth (TSB) at 37 °C for 10 h. Then, each solution was measured spectrometrically at 600 nm (*A*_600 nm_) and compared with the value of the control. The control was TSB without each treatment. In order to evaluate the effect of magnesium, we performed the same experiments without magnesium. The values are the mean ± SEM from three independent experiments for *P*. *aeruginosa* (NBRC 13275) and MRSA. *p<0.05, **p<0.01 (unpaired t-test). LYZOX: lysozyme-chitosan oligosaccharide conjugate. COS: chitosan oligosaccharide. Mg: magnesium.

For confirmation of the chelating properties of LYZOX, we performed a growth inhibition test of LYZOX with or without magnesium ions as described previously [29]. In *P*. *aeruginosa* (NBRC 13275) (Fig 9a), magnesium significantly inhibited the antibacterial activities of LYZOX and COS (LYZOX [10,000 μg/mL] with magnesium vs without magnesium, p<0.001; COS [10,000 μg/mL] with magnesium vs without magnesium, p<0.001). In MRSA (Fig 9b), magnesium significantly inhibited the antibacterial activities of LYZOX and COS (LYZOX [10,000 μg/mL] with magnesium vs without magnesium, p = 0.032; COS [10,000 μg/mL] with magnesium vs without magnesium, p = 0.004).

### Comparison of the minimal inhibitory concentrations

To compare the antibacterial activity of LYZOX, the mixture and the conventional antibiotics, the MICs of these for *P*. *aeruginosa*, *A*. *baumannii* and MRSA were compared. The MIC of the mixture (lysozyme and COS) was higher than the MIC of LYZOX for each type of bacteria (*P*. *aeruginosa* [NBRC 13275]: LYZOX 400 μg/mL, mixture 2,000 μg/mL; *P*. *aeruginosa* [PAO1]: LYZOX 200 μg/mL, mixture 2,000 μg/mL; *A*. *baumannii*: LYZOX 100 μg/mL, mixture 800 μg/mL; MRSA: LYZOX 25 μg/mL, mixture 250 μg/mL) (Table 1). The MIC of LYZOX was higher than the MIC of colistin in *P*. *aeruginosa* and *A*. *baumannii* (*P*. *aeruginosa* [NBRC 13275]: LYZOX 400 μg/mL, colistin 0.25 μg/mL; *P*. *aeruginosa* [PAO1]: LYZOX 200 μg/mL, colistin 1.0 μg/mL; *A*. *baumannii*: LYZOX 100 μg/mL, colistin 0.25 μg/mL) and was higher than the MIC of vancomycin in MRSA (LYZOX 25 μg/mL, vancomycin 0.25 μg/mL). It has been reported that vancomycin is not effective against gram-negative bacteria, and colistin is not effective against gram-positive bacteria [38, 39]. In contrast, LYZOX and the mixture were effective against each type of bacteria.

**Table 1:** Comparison of the minimal inhibitory concentrations.

	MIC (μg/mL)			
	LYZOX	Mixture	Colistin	Vancomycin
*Pseudomonas aeruginosa* (NBRC13275)	400	2,000	0.25	R
*Pseudomonas aeruginosa* (PAO1)	200	2,000	1.0	R
*Acinetobacter baumannii* (JCM6841)	100	800	0.25	R
MRSA (IID1677)	25	250	R	0.25

R: resistance of antibiotics

The mixture comprised lysozyme and chitosan oligosaccharide (COS) at a weight ratio of 1:1. For example, 2,000 μg/mL mixture was composed of 1,000 μg/mL lysozyme and 1,000 μg/mL COS.

### Acquired drug resistance test

**Fig 10:**
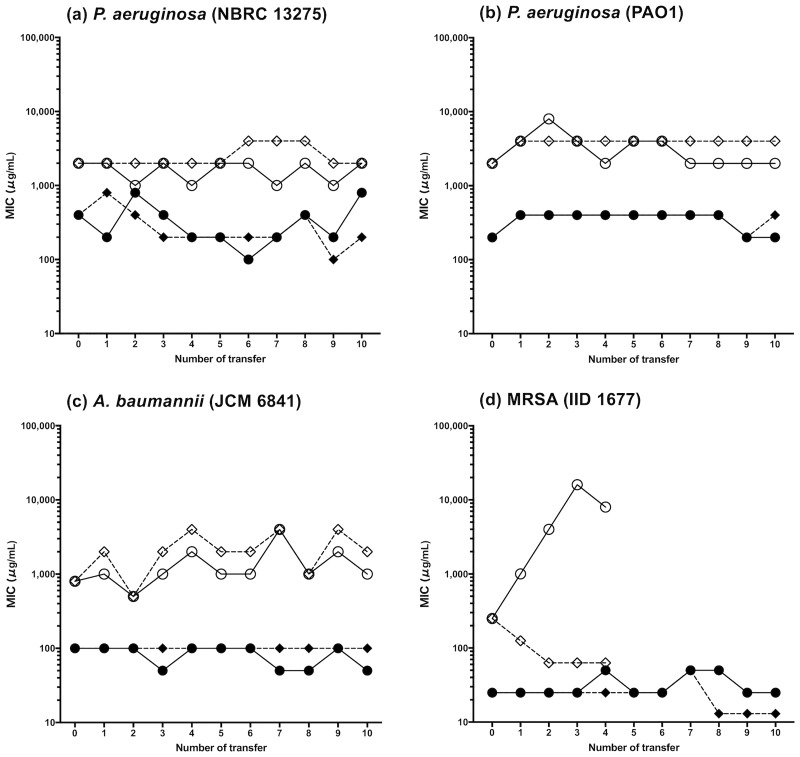
Acquired drug resistance test. Bacteria were subcultured repeatedly in LB broth containing lysozyme-chitosan oligosaccharide conjugate (LYZOX) or the mixture (lysozyme and chitosan oligosaccharide). They were evaluated for their change of susceptibility to LYZOX or mixture. The control was not subcultured. (a) *Pseudomonas aeruginosa* (NBRC 13275), (b) *Pseudomonas aeruginosa* (PAO1), (c) *Acinetobacter baumannii* (JCM 6841), (d) MRSA (IID 1677). For MRSA, the test of mixture was finished because antibacterial resistance of the mixture was confirmed at the fourth transfer. Symbols: filled circles, LYZOX, subcultured; filled rhombuses, LYZOX, control; open circles, mixture, subcultured; open rhombuses, mixture, control.

Acquired drug resistance tests are shown in Fig 10. *P*. *aureginosa* and *A*. *baumannii* did not acquire resistance to LYZOX and the mixture, although the bacteria were subcultured for 10 passages. By contrast, MRSA acquired resistance to the mixture at the second transfer when the MIC was quadruple the first MIC. We finished the test of the mixture at the fourth transfer because antibacterial resistance to the mixture was confirmed. In contrast, MRSA also did not acquire resistance to LYZOX.

### Hemolytic toxicity test

LYZOX produced negligible hemolytic rates in rabbit red blood cells after 1 h of incubation at 37°C (Table 2). The hemolytic rate of LYZOX was less than 5% at a concentration ranging from 10 μg/mL to 10,000 μg/mL. A rate of 5% hemolysis or less has been reported to be permissible and safe for clinical biomaterials [33].

**Table 2:** Hemolytic toxicity test.

Concentration of LYZOX	AS (OD_545 nm_)	AP (OD_545 nm_)	AN (OD_545 nm_)	HR (%)
10,000 μg/mL	0.489±0.005	1.458±0.022	0.457±0.012	2.17±0.64
1,000 μg/mL	0.155±0.008	1.161±0.013	0.130±0.006	2.19±0.26
100 μg/mL	0.097±0.004	1.091±0.010	0.086±0.007	1.05±0.70
10 μg/mL	0.090±0.010	1.027±0.020	0.084±0.009	0.55±0.26

The optical density (OD) values at 545 nm were expressed as the mean and SEM of triplicate measurements.

AS: absorbance of the sample.

AP: absorbance of the positive control.

AN: absorbance of the negative control.

HR: hemolytic rate.

### Heat stability of lysozyme and LYZOX

**Fig 11:**
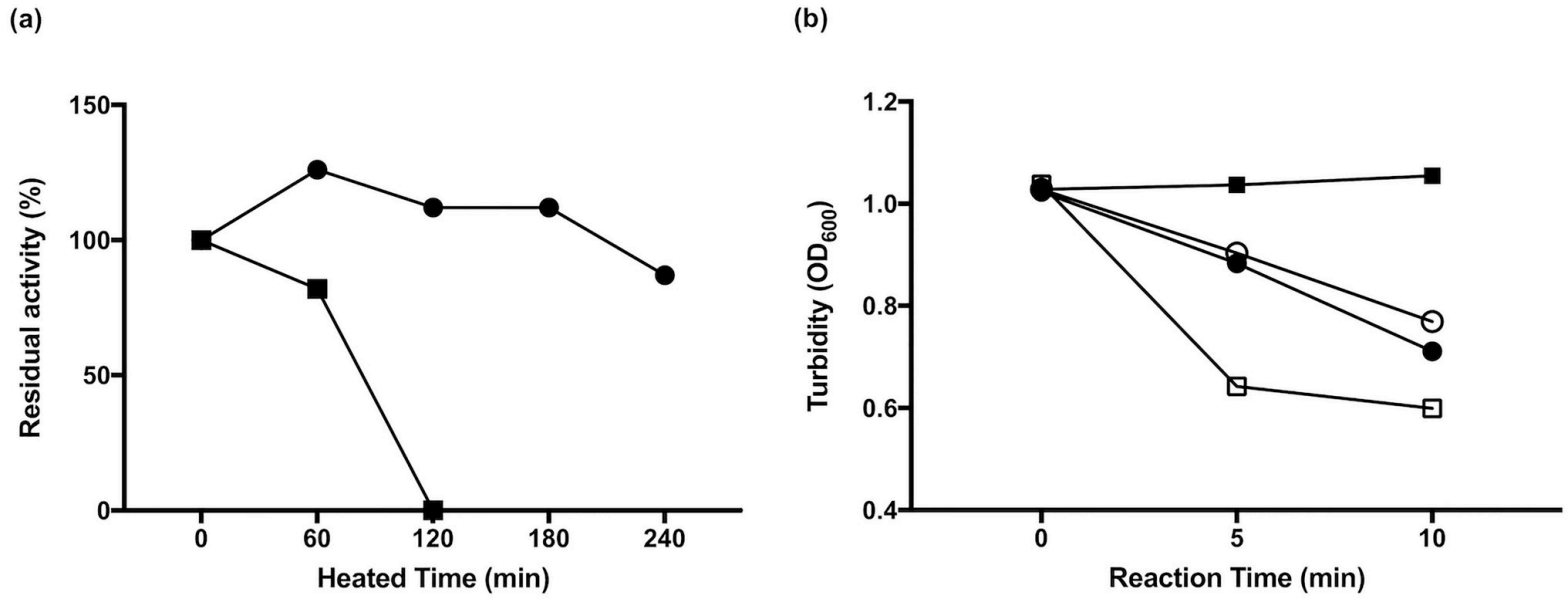
Heat stability of lysozyme and LYZOX. (a) The effects of heat were tested with lysozyme-chitosan oligosaccharide (LYZOX) and lysozyme exposed to 80°C for various times. The lytic activity of heated agents against *Micrococcus luteus* for 10 min were measured at OD_600_. The values are the per cent of residual activity. Symbols: filled circles, LYZOX, filled squares, lysozyme. (b) Comparison of lytic activity of LYZOX and lysozyme for 120 min at 80°C. Lytic activity against *Micrococcus luteus* was measured at OD_600_. Symbols: open circles, non-heated LYZOX; filled circles, heated LYZOX; open squares, non-heated lysozyme; filled squares, heated lysozyme.

The effect of LYZOX on heat stability is shown in Fig 11. Fig 11a shows that LYZOX and lysozyme were exposed to 80°C for various times. Although the lysozyme lost lytic activity by heating for 120 min, the LYZOX retained its lytic activity independent of the heating time. Fig 11b shows a comparison of lytic activity for the reaction time of LYZOX and lysozyme. Lysozyme without heat treatment showed lytic activity, whereas lysozyme with heat treatment lost lytic activity. Although LYZOX lysed *Micrococcus luteus* more slowly than lysozyme without heat treatment, heat treatment did not cause the loss of the lytic activity of LYZOX.

## Discussion

The aim of the present study was to evaluate the antibacterial activity of LYZOX against *P*. *aeruginosa*, *A*. *baumannii* and MRSA and to determine the antibacterial mechanism of LYZOX. We demonstrated that LYZOX exhibited superior antibacterial activity compared to its components against these bacteria, and the cell membrane integrity assay, NPN assay, and ONPG assay results and the CLSM and electron microscopy findings demonstrate that LYZOX affected the bacterial membranes, leading to membrane disruption and the release of intracellular contents.

LYZOX is a lysozyme-COS conjugate produced by the Maillard reaction. Lysozyme is an AMP that is involved in innate immunity [5, 8]. As lysozyme is easily obtained in a purified form from chicken eggs [40], it has been used in foods and pharmaceutical products because of its antimicrobial effects. However, there are some problems associated with its use because of its heat instability and its narrow spectrum of antimicrobial activity [8]. The raw material used for the production of chitosan is chitin, which is obtained mainly from the shells of crustaceans, such as crabs and shrimp [9, 10]. Although chitosan has a broad spectrum of antimicrobial activity and lower toxicity towards mammalian cells [10], its insolubility in aqueous solutions at physiological pH limits its application. Previous studies showed that polysaccharides conjugated to lysozyme via the Maillard reaction acquired heat stability, increased solubility, emulsifying activity and a wide antibacterial spectrum [7, 16, 17]. Kato et al reported that the antibacterial activity of dextran-lysozyme conjugates was effective against gram-positive and gram-negative bacteria, such as *Vibrio*, *Aeromonas*, *Proteus*, *Klebsiella* and *E*. *coli* [7], whereas the antibacterial activity of chitosan-lysozyme conjugates was shown only against *E*. *coli* [7, 16]. There have been no reports that have evaluated the antibacterial activity of chitosan-lysozyme conjugates against *P*. *aeruginosa*, *A*. *baumannii* and MRSA. In the present study, we confirmed that LYZOX was an MRP on the basis of increases in fluorescence intensity and molecular weight during SDS-PAGE, which were similar to previously reported results [16, 20]. Then, we assessed the antibacterial activity of LYZOX. LYZOX killed 50% more *P*. *aeruginosa* (NBRC 13275), *A*. *baumannii* and MRSA than the control treatment after 60 min. LYZOX was more effective than the control treatment against *P*. *aeruginosa* (PAO1) after 120 min. LYZOX showed greater inhibition of bacterial growth in *P*. *aeruginosa*, *A*. *baumannii* and MRSA compared with its components. We confirmed that LYZOX showed antibacterial activity against these bacteria, and these results suggested that LYZOX had enhanced antibacterial effects when compared to lysozyme and/or COS.

We examined the antibacterial mechanism of LYZOX. Although lysozyme is able to hydrolyze peptidoglycan, the hydrophobic outer membranes of gram-negative bacteria generally disrupt lysozyme activity [8]. Warren et al reported that acetone improved the sensitivity of *P*. *aeruginosa* to lysozyme because of its fat-solvent action against certain lipids in the cell wall [41]. Emulsifiers, such as deoxycholate solution or X-100, could solubilize gram-negative outer membrane [42, 43]. It was possible that the emulsifying ability, which was acquired through the Maillard reaction with COS, destroyed the outer membrane and that glycosidase of LYZOX was able to hydrolyze peptidoglycan of gram-negative bacteria. However, LGC did not have antibacterial activity against *P*. *aeruginosa* and *A*. *baumannii* as strong as that of LYZOX. It was thought that the antimicrobial action of LYZOX was a result of not only its emulsifying ability and the glycosidase action of lysozyme but also the antibacterial mechanism of chitosan itself. Although the mechanism of the antibacterial action of chitosan has not yet been fully elucidated, it is presumed to be due to its interactions with the bacterial surface. Four potential models for this mechanism have been proposed [9]. The first model, which has been widely accepted, involves an electrostatic attraction between the cationic groups in chitosan and the negatively charged components on the bacterial surface. The second model suggests that chitosan induces changes in the permeability of the outer envelope in gram-negative bacteria, which results in membrane vesicles on their outer surfaces [27, 44]. In the third model, chitosan penetrates microbial cells and interacts with their DNA. The fourth model proposes that chitosan acts as a chelating agent that binds metal ions such as magnesium and calcium. Divalent cations stabilize bacterial cell membranes and are critical for the functioning of various membrane-bound enzymes [24].

AMPs, including lysozyme, have a common structural feature that is referred to as facial amphiphilicity, in which cationic side chains and lipophilic side chains are segregated [28]. COS has cationic properties, and the Maillard reaction enhanced its emulsifying properties (amphiphilicity) [7]. LYZOX might obtain enhanced cationic properties and facial amphiphilicity through the Maillard reaction. In addition, as shown by the SDS-PAGE analysis, the increase in molecular weight in LYZOX might be a result of clustered MRPs, which were polymerized via the Maillard reaction. Rahman et al reported that a class of cationic antimicrobial polymers that were designed to cluster local facial amphiphilicity from repeating units had enhanced interactions with bacterial membranes [28]. We speculated that LYZOX enhanced interactions with bacterial membranes not only by increasing the cationic properties and the amphiphilicity but also by clustering MRPs to produce cationic antimicrobial polymers. The cell membrane integrity assays showed that LYZOX affected bacterial cell walls. In gram-negative bacteria (*P*. *aeruginosa* and *A*. *baumannii*), the NPN assays showed an increase in outer membrane permeability, and the ONPG assays showed an increase in inner membrane permeability. In gram-positive bacteria (MRSA), the NPN and ONPG assays showed an increase in plasma membrane permeability. CLSM revealed that LYZOX damaged the membranes of each type of bacteria, which was consistent with the results of the NPN and ONPG assays. CLSM could show that bacterial cells with/without damage membrane were aggregated for each type of bacteria. A previous study reported that the aggregation of bacterial cells under the influence of chitosan might have contributed to growth inhibition [45]. The aggregation of bacterial cells treated with LYZOX might be related to growth inhibition. In gram-negative bacteria, electron micrographs revealed that the bacteria treated with LYZOX had the morphological features of both lysozyme-treated and COS-treated bacteria, which were characterized by spherical cells with outer membrane vesicles. We speculated that the outer membranes of gram-negative bacteria were damaged not only because of the emulsifying ability of LYZOX but also because of the increase in outer membrane permeability caused by the chitosan component in LYZOX, and the glycosidase activity of LYZOX might have been able to hydrolyze the peptidoglycans inside the outer membranes of gram-negative bacteria. Furthermore, TEM revealed that the bacterial cell walls were destroyed, and the intracellular contents were released from the cell. In gram-positive bacteria (MRSA) treated with LYZOX, TEM micrographs revealed that the bacterial cell walls were thickened and destroyed. Because TEM revealed that MRSA cells treated with lysozyme and COS were disrupted, it was speculated that, LYZOX disrupted bacterial cell membranes in gram-positive bacteria due to the combined effects of lysozyme and COS, which led to the leakage of the intracellular contents. Finally, we confirmed that LYZOX had a chelating property that bound metal ions on the basis of the reduction in antibacterial activity upon the addition of magnesium to the LYZOX solution. This chelating property, particularly in *P*. *aeruginosa*, was considered to be one of the antibacterial mechanisms of LYZOX. As described above, LYZOX had multiple mechanisms and properties that contributed to its antibacterial activity, including cationic properties, emulsifying properties (amphiphilicity), membrane disruption activity (disruption of the outer membrane and the plasma membrane [inner membrane]) and chelating properties.

To compare the antibacterial activity of LYZOX with that of conventional antibiotics, the MIC of LYZOX were compared with those of colistin and vancomycin in each type of bacteria. The MIC of colistin in gram-negative bacteria and the MIC of vancomycin in MRSA were lower than the MIC of LYZOX in these bacteria. It is known that vancomycin is not efficient against gram-negative bacteria due to its large molecular size and inability to penetrate the outer bacterial membrane [38] and that polymyxins, including colistin, are not active against gram-positive bacteria [39]. In contrast, LYZOX had a broader spectrum of antibacterial activity than colistin and vancomycin.

We evaluated the antibacterial resistance to LYZOX for *P*. *aeruginosa*, *A*. *baumannii* and MRSA by acquired drug resistance tests. *P*. *aeruginosa* and *A*. *baumannii* did not acquire resistance against LYZOX and the mixture, whereas the MIC of the mixture was higher than that of LYZOX in these bacteria. MRSA also did not acquire resistance against LYZOX, and the MIC of the mixture was higher than that of LYZOX in MRSA, similar to gram-negative bacteria. MRSA acquired resistance against the mixture. There was no bactericidal activity of lysozyme against MRSA, although COS had bactericidal activity as almost equal to LYZOX (S4 Fig). The isoelectric point of chitosan was reported to be approximately 6.5, whereas the isoelectric point of lysozyme is approximately 11.0 [9]. Therefore, lysozyme has greater cationic charge on its surface than chitosan because of its isoelectric point. Because of this, we suggest that, in the mixture, lysozyme adhered to bacterial walls and then interfered with the antibacterial action of COS against MRSA. We speculated that the multiple mechanisms and properties were associated with low drug resistance.

We evaluated the characteristics of LYZOX. LYZOX has little toxicity because lysozyme and chitosan are made from natural products, and the Maillard reaction does not require any chemicals or enzymes. In the present study, a hemolytic toxicity test showed the low hemolytic activity of LYZOX. Previous reports showed that the turbidity of native lysozyme was significantly higher than that of lysozyme-glucosamine conjugates above 60°C and that of LGC at 95°C, which suggested that lysozyme-polysaccharide conjugates had increased heat stability [7, 17]. Although there have been no reports that have evaluated the heat stability of lysozyme-COS conjugates, the present study confirmed that LYZOX maintained its antibacterial activity regardless of the heating time at 80°C. HMC-lysozyme conjugates were difficult to use for treatment due to their water insolubility compared to low molecular weight chitosan-lysozyme conjugates, which had excellent solubility over a wide range of pH values [16]. Chung et al showed that a water-soluble chitosan conjugate had relatively high antibacterial activity, regardless of pH, against various microorganisms compared to an acid-soluble chitosan [46]. Therefore, we used a water-soluble chitosan, COS, as the partner saccharide in the Maillard reaction with lysozyme. LYZOX might be expected to be more easily used in pharmaceuticals than either lysozyme and chitosan because of its low toxicity, heat stability and solubility at neutral pH.

In conclusion, LYZOX had antibacterial activity against *P*. *aeruginosa*, *A*. *baumannii*, and MRSA. Additionally, these bacteria did not acquire resistance to LYZOX. The multiple mechanisms underlying the effects of LYZOX could be involved in its antibacterial activity and low drug resistance against these bacteria. LYZOX has versatility because of its excellent heat stability, solubility and low toxicity. LYZOX could be a new drug candidate that could be used for the control of refractory infections that would lead to a reduction in medical expenses.

## Supporting information

S1 Fig Growth inhibition test for various concentrations of lysozyme-chitosan oligosaccharide conjugates in *Pseudomonas aeruginosa*. *P*. *aeruginosa* (NBRC 13275) incubated with various concentrations of lysozyme-chitosan oligosaccharide conjugates (LYZOX) solution in tryptic soy broth at 37°C for 3, 6, 9, 12 and 24 h. The dilutions were plated, the colonies were counted following growth overnight, and results were compared with the control. The values are the mean ± SEM from 11 independent experiments. Symbols: circles, LYZOX (10,000 μg/mL); squares, LYZOX (2,000 μg/mL); up-pointing triangles, LYZOX (1,000 μg/mL); down-pointing triangles, LYZOX (200 μg/mL).
(TIFF)

S2 Fig Results of Maillard reaction with lysozyme and chitosan oligosaccharide. (a) Changes in fluorescence intensity produced by the Maillard reaction. The fluorescence intensity of the lysozyme-chitosan oligosaccharide conjugate (LYZOX) solution (500 μg/mL) and the mixture (lysozyme [250 μg/mL] and COS [250 μg/mL]) were measured (excitation: 370 nm/emission 440 nm). The values are the mean ± SEM of triplicate measurements. **p<0.01 (unpaired t-test). (b) Sodium dodecyl sulfate-polyacrylamide gel electrophoresis analysis with a 10–20% gradient gel. Ten microliters of LYZOX (500 μg/mL), lysozyme (250 μg/mL) or the mixture (lysozyme [250 μg/mL] and COS [250 μg/mL]) were loaded into each well. M, molecular weight marker; lane 1, lysozyme; lane 2, mixture (lysozyme and chitosan oligosaccharide); lane 3, lysozyme-chitosan oligosaccharide conjugate (LYZOX).
(TIFF)

S3 Fig Assays of bactericidal activity for various concentrations of lysozyme-chitosan oligosaccharide conjugates in *Pseudomonas aeruginosa*. *P*. *aeruginosa* (NBRC 13275) was incubated with various concentrations of lysozyme-chitosan oligosaccharide conjugates (LYZOX) in saline at 37°C in a water bath for 0 min, 60 min and 120 min. The dilutions were plated, and the colonies were counted following growth overnight. The values are the mean ± SEM from three independent experiments. Symbols: circles, saline; down-pointing triangles, LYZOX (200 μg/mL); up-pointing triangles, LYZOX (2,000 μg/mL); squares, LYZOX (10,000 μg/mL).
(TIFF)

S4 Fig Assays of bactericidal activity for various treatments against MRSA. MRSA (IID 1677) was incubated with each treatment solution in saline at 37°C in a water bath for 0 min, 60 min and 120 min. Treatments were lysozyme-chitosan oligosaccharide conjugates (LYZOX) solution (2,000 μg/mL), chitosan oligosaccharide (COS) solution (1,000 μg/mL), lysozyme (1,000 μg/mL) and mixed solution (lysozyme [1,000 μg/mL] and COS [1,000 μg/mL]). The dilutions were plated, and the colonies were counted following growth overnight. The values are the mean ± SEM from four independent experiments. Symbols: circles, saline; squares, LYZOX; up-pointing triangles, COS; down-pointing triangles, lysozyme; rhombuses, mixture. *p<0.05 or **p<0.01 compared with saline; ††p<0.01 compared with lysozyme. (unpaired t-test).
(TIFF)

S1 Table Minimal inhibitory concentrations of chitosan or modified chitosan in previous reports. MIC: minimal inhibitory concentration. HMC: high molecular weight chitosan (molecular weight [MW] of 624 kDa). LMC: low molecular weight chitosan (MW of 107 kDa). CM: chitosan microparticles. HTCCs: N-(2-hydroxypropyl)-3-trimethylammonium chitosan chloride. HTCCs are water-soluble derivatives of chitosan (CS) that are synthesized by a reaction between glycidyl-trimethyl-ammonium chloride and CS. Six different polymers with different degrees of quaternization and different molecular weights were synthesized as HTTCs. N.D.: no data. Clinical isolate: CI. NDM: New Delhi metallo-beta lactamase.
(DOCX)

## References

[1] Ventola CL. The antibiotic resistance crisis: part 1: causes and threats. P T. 2015;40(4):277–83. Epub 2015/04/11. . 25859123 PMC4378521

[2] Potron A, Poirel L, Nordmann P. Emerging broad-spectrum resistance in Pseudomonas aeruginosa and Acinetobacter baumannii: Mechanisms and epidemiology. Int J Antimicrob Agents. 2015;45(6):568–85. Epub 2015/04/11. 10.1016/j.ijantimicag.2015.03.001 . 25857949

[3] Paterson GK, Harrison EM, Holmes MA. The emergence of mecC methicillin-resistant Staphylococcus aureus. Trends Microbiol. 2014;22(1):42–7. Epub 2013/12/18. 10.1016/j.tim.2013.11.003 . 24331435 PMC3989053

[4] J ON. Tackling drug-resistant infections globally: final report and recommendations 2016. https://amr-review.org/sites/default/files/160518_Final%20paper_with%20cover.pdf.

[5] Travis SM, Singh PK, Welsh MJ. Antimicrobial peptides and proteins in the innate defense of the airway surface. Curr Opin Immunol. 2001;13(1):89–95. Epub 2001/01/13. . 11154923 10.1016/s0952-7915(00)00187-4

[6] Wang J, Dou X, Song J, Lyu Y, Zhu X, Xu L, et al Antimicrobial peptides: Promising alternatives in the post feeding antibiotic era. Med Res Rev. 2018 Epub 2018/10/26. 10.1002/med.21542 . 30353555

[7] Kato A. Industrial applications of Maillard-type protein-polysaccharide conjugates. Food Science and Technology Research. 2002;8(3):193–9. 10.3136/fstr.8.193

[8] Aminlari L, Hashemi MM, Aminlari M. Modified Lysozymes as Novel Broad Spectrum Natural Antimicrobial Agents in Foods. J Food Sci. 2014;79(6):R1077–R90. 10.1111/1750-3841.12460 24837015

[9] Sahariah P, Masson M. Antimicrobial Chitosan and Chitosan Derivatives: A Review of the Structure-Activity Relationship. Biomacromolecules. 2017;18(11):3846–68. Epub 2017/09/22. 10.1021/acs.biomac.7b01058 . 28933147

[10] Cheung RC, Ng TB, Wong JH, Chan WY. Chitosan: An Update on Potential Biomedical and Pharmaceutical Applications. Mar Drugs. 2015;13(8):5156–86. Epub 2015/08/20. 10.3390/md13085156 . 26287217 PMC4557018

[11] Costa EM, Silva S, Tavaria FK, Pintado MM. Insights into chitosan antibiofilm activity against methicillin-resistant Staphylococcus aureus. J Appl Microbiol. 2017;122(6):1547–57. Epub 2017/04/04. 10.1111/jam.13457 . 28370752

[12] Ma Z, Kim D, Adesogan AT, Ko S, Galvao K, Jeong KC. Chitosan Microparticles Exert Broad-Spectrum Antimicrobial Activity against Antibiotic-Resistant Micro-organisms without Increasing Resistance. ACS Appl Mater Interfaces. 2016;8(17):10700–9. Epub 2016/04/09. 10.1021/acsami.6b00894 . 27057922

[13] Hoque J, Adhikary U, Yadav V, Samaddar S, Konai MM, Prakash RG, et al Chitosan Derivatives Active against Multidrug-Resistant Bacteria and Pathogenic Fungi: In Vivo Evaluation as Topical Antimicrobials. Mol Pharm. 2016;13(10):3578–89. Epub 2016/09/03. 10.1021/acs.molpharmaceut.6b00764 . 27589087

[14] Costa EM, Silva S, Veiga M, Vicente S, Tavaria FK, Pintado ME. Investigation of chitosan’s antibacterial activity against vancomycin resistant microorganisms and their biofilms. Carbohydr Polym. 2017;174:369–76. Epub 2017/08/20. 10.1016/j.carbpol.2017.06.087 . 28821080

[15] Costa EM, Silva S, Vicente S, Veiga M, Tavaria F, Pintado MM. Chitosan as an effective inhibitor of multidrug resistant Acinetobacter baumannii. Carbohydr Polym. 2017;178:347–51. Epub 2017/10/21. 10.1016/j.carbpol.2017.09.055 . 29050604

[16] Song Y, Babiker EE, Usui M, Saito A, Kato A. Emulsifying properties and bactericidal action of chitosan-lysozyme conjugates. Food Research International. 2002;35(5):459–66. 10.1016/S0963-9969(01)00144-2

[17] Ramezani R, Esmailpour M, Aminlari M. Effect of conjugation with glucosamine on the functional properties of lysozyme and casein. Journal of the Science of Food and Agriculture. 2008;88(15):2730–7. 10.1002/jsfa.3400

[18] Nakamura S, Gohya Y, Losso JN, Nakai S, Kato A. Protective effect of lysozyme-galactomannan or lysozyme-palmitic acid conjugates against Edwardsiella tarda infection in carp, Cyprinus carpio L. FEBS Lett. 1996;383(3):251–4. Epub 1996/04/01. . 8925907 10.1016/0014-5793(96)00260-8

[19] Nakamura S, Kato A. Multi-functional biopolymer prepared by covalent attachment of galactomannan to egg-white proteins through naturally occurring Maillard reaction. Nahrung. 2000;44(3):201–6. 10.1002/1521-3803(20000501)44:3<201::AID-FOOD201>3.0.CO;2-S 10907243

[20] Yang JE, Chun SH, Kim HH, Choi HD, Lee KW. Characterization of Maillard-type lysozyme-galactomannan conjugate having immune-enhancing effects. Food Chem. 2017;227:149–57. Epub 2017/03/10. 10.1016/j.foodchem.2017.01.076 . 28274415

[21] Chen LC, Chiang WD, Chen WC, Chen HH, Huang YW, Chen WJ, et al Influence of alanine uptake on Staphylococcus aureus surface charge and its susceptibility to two cationic antibacterial agents, nisin and low molecular weight chitosan. Food Chem. 2012;135(4):2397–403. 10.1016/j.foodchem.2012.06.122 22980819

[22] Kumar ABV, Varadara MC, Gowda LR, Tharanathan RN. Characterization of chito-oligosaccharides prepared by chitosanolysis with the aid of papain and Pronase, and their bactericidal action against Bacillus cereus and Escherichia coli. Biochem J. 2005;391:167–75. 10.1042/BJ20050093 15932346 PMC1276913

[23] Xing K, Chen XG, Kong M, Liu CS, Cha DS, Park HJ. Effect of oleoyl-chitosan nanoparticles as a novel antibacterial dispersion system on viability, membrane permeability and cell morphology of Escherichia coli and Staphylococcus aureus. Carbohyd Polym. 2009;76(1):17–22. 10.1016/j.carbpol.2008.09.016

[24] Chen CZ, Cooper SL. Interactions between dendrimer biocides and bacterial membranes. Biomaterials. 2002;23(16):3359–68. Epub 2002/07/09. . 12099278 10.1016/s0142-9612(02)00036-4

[25] Helander IM, Mattila-Sandholm T. Fluorometric assessment of gram-negative bacterial permeabilization. J Appl Microbiol. 2000;88(2):213–9. Epub 2000/03/29. . 10735988 10.1046/j.1365-2672.2000.00971.x

[26] Han HM, Gopal R, Park Y. Design and membrane-disruption mechanism of charge-enriched AMPs exhibiting cell selectivity, high-salt resistance, and anti-biofilm properties. Amino Acids. 2016;48(2):505–22. Epub 2015/10/10. 10.1007/s00726-015-2104-0 . 26450121

[27] Liu H, Du YM, Wang XH, Sun LP. Chitosan kills bacteria through cell membrane damage. Int J Food Microbiol. 2004;95(2):147–55. 10.1016/j.ijfoodmicro.2004.01.022 15282127

[28] Rahman MA, Bam M, Luat E, Jui MS, Ganewatta MS, Shokfai T, et al Macromolecular-clustered facial amphiphilic antimicrobials. Nat Commun. 2018;9(1):5231 Epub 2018/12/12. 10.1038/s41467-018-07651-7 . 30531920 PMC6286373

[29] Kong M, Chen XG, Liu CS, Liu CG, Meng XH, Yu le J. Antibacterial mechanism of chitosan microspheres in a solid dispersing system against E. coli. Colloids Surf B Biointerfaces. 2008;65(2):197–202. Epub 2008/05/30. 10.1016/j.colsurfb.2008.04.003 . 18508247

[30] M07-A10. Methods for Dilution Antimicrobial Tests for Bacteria that Grow Aerobically.: Approved Standard. Tenth ed. Wayne, PA: Clinical and Laboratory Standards Institute; 2015.

[31] Alekshun MN, Levy SB. Molecular mechanisms of antibacterial multidrug resistance. Cell. 2007;128(6):1037–50. 10.1016/j.cell.2007.03.004 17382878

[32] Fujimura T, Kimura Y, Yoshida I, Higashiyama I, Jinushi Y, Munekage T, et al In vitro antibacterial activity of doripenem, a novel parenteral carbapenem. Jpn J Chemother. 2005;53(S-1):57–70.

[33] Deng JD, He B, He DH, Chen ZF. A potential biopreservative: Chemical composition, antibacterial and hemolytic activities of leaves essential oil from Alpinia guinanensis. Ind Crop Prod. 2016;94:281–7. 10.1016/j.indcrop.2016.09.004

[34] Saito A, Sako Y, Usui M, Azakami H, Kato A. Functional properties of glycosylated lysozyme secreted in Pichia pastoris. Biosci Biotechnol Biochem. 2003;67(11):2334–43. Epub 2003/12/04. 10.1271/bbb.67.2334 . 14646191

[35] Lee J, Kim I, Yeo S, Kim D, Kim M. Dextran-Conjugated Lysozymes Inhibit the Growth of Shigella sonnei and Viral Hemorrhagic Septicemia Virus. Prev Nutr Food Sci. 2018;23(1):60–9. Epub 2018/04/18. 10.3746/pnf.2018.23.1.60 . 29662849 PMC5894787

[36] Xu C, Li JL, Yang LQ, Shi F, Yang L, Ye M. Antibacterial activity and a membrane damage mechanism of Lachnum YM30 melanin against Vibrio parahaemolyticus and Staphylococcus aureus. Food Control. 2017;73:1445–51. 10.1016/j.foodcont.2016.10.048

[37] Shen SX, Zhang TH, Yuan Y, Lin SY, Xu JY, Ye HQ. Effects of cinnamaldehyde on Escherichia coli and Staphylococcus aureus membrane. Food Control. 2015;47:196–202. 10.1016/j.foodcont.2014.07.003

[38] Fernandes MM, Ivanova K, Hoyo J, Perez-Rafael S, Francesko A, Tzanov T. Nanotransformation of Vancomycin Overcomes the Intrinsic Resistance of Gram-Negative Bacteria. ACS Appl Mater Interfaces. 2017;9(17):15022–30. Epub 2017/04/11. 10.1021/acsami.7b00217 . 28393523

[39] Poirel L, Jayol A, Nordmann P. Polymyxins: Antibacterial Activity, Susceptibility Testing, and Resistance Mechanisms Encoded by Plasmids or Chromosomes. Clin Microbiol Rev. 2017;30(2):557–96. Epub 2017/03/10. 10.1128/CMR.00064-16 . 28275006 PMC5355641

[40] Mine Y, Yang M. Recent advances in the understanding of egg allergens: basic, industrial, and clinical perspectives. J Agric Food Chem. 2008;56(13):4874–900. Epub 2008/06/12. 10.1021/jf8001153 . 18543935

[41] Warren GH, Gray J, Bartell P. The Lysis of Pseudomonas-Aeruginosa by Lysozyme. Journal of Bacteriology. 1955;70(5):614–9. 13271300 10.1128/jb.70.5.614-619.1955 PMC357720

[42] Nakamura K, Mizushima S. In vitro reassembly of the membranous vesicle from Escherichia coli outer membrane components. Role of individual components and magnesium ions in reassembly. Biochim Biophys Acta. 1975;413(3):371–93. Epub 1975/12/16. . 1103979 10.1016/0005-2736(75)90122-4

[43] DePamphilis ML. Dissociation and reassembly of Escherichia coli outer membrane and of lipopolysaccharide, and their reassembly onto flagellar basal bodies. J Bacteriol. 1971;105(3):1184–99. Epub 1971/03/01. . 4100836 10.1128/jb.105.3.1184-1199.1971 PMC248550

[44] Helander IM, Nurmiaho-Lassila EL, Ahvenainen R, Rhoades J, Roller S. Chitosan disrupts the barrier properties of the outer membrane of Gram-negative bacteria. Int J Food Microbiol. 2001;71(2–3):235–44. 10.1016/S0168-1605(01)00609-2 11789941

[45] Tang H, Zhang P, Kieft TL, Ryan SJ, Baker SM, Wiesmann WP, et al Antibacterial action of a novel functionalized chitosan-arginine against Gram-negative bacteria. Acta Biomater. 2010;6(7):2562–71. Epub 2010/01/12. 10.1016/j.actbio.2010.01.002 . 20060936 PMC2874111

[46] Chung YC, Yeh JY, Tsai CF. Antibacterial characteristics and activity of water-soluble chitosan derivatives prepared by the Maillard reaction. Molecules. 2011;16(10):8504–14. Epub 2011/10/13. 10.3390/molecules16108504 . 21989311 PMC6264222

