# Supplementary figures and images for: Antibacterial activity of lysozyme-chitosan oligosaccharide conjugates (LYZOX) against Pseudomonas aeruginosa, Acinetobacter baumannii and Methicillin-resistant Staphylococcus aureus
Source: PLoS One. 2019 May 28;14(5):e0217504. doi: 10.1371/journal.pone.0217504 (PMC6538184; doi:10.1371/journal.pone.0217504)

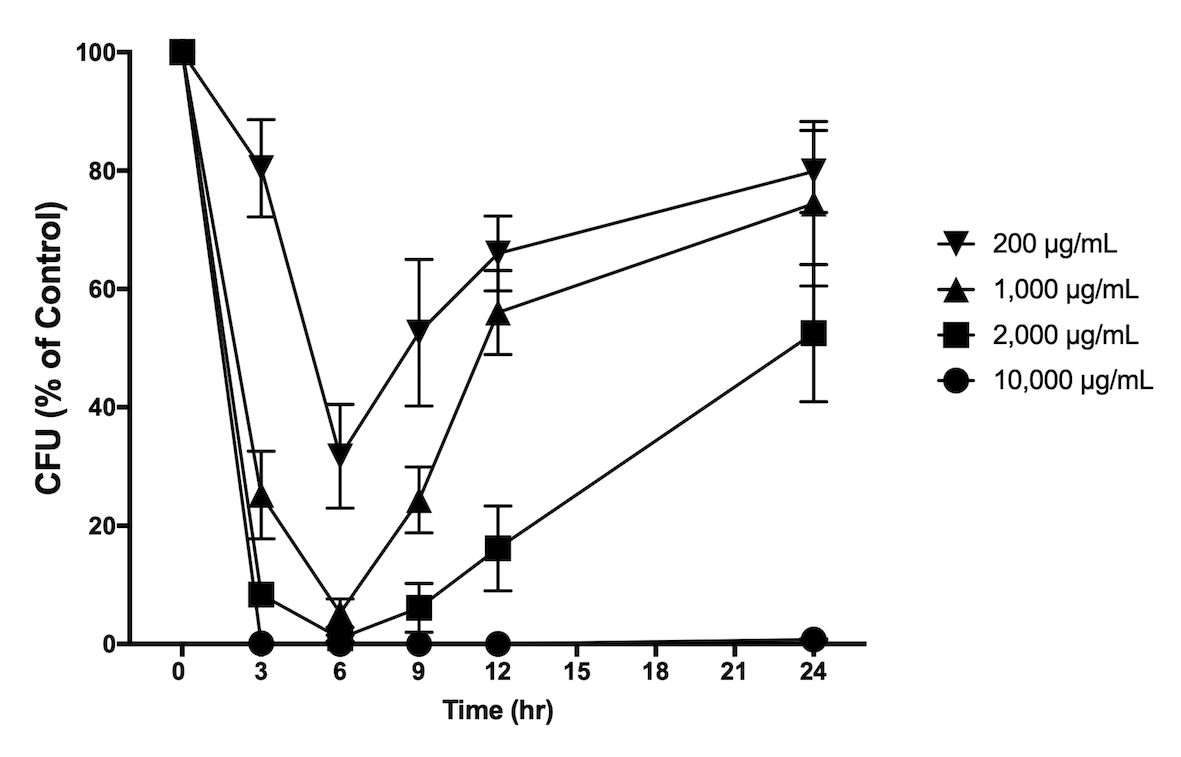

Supplement: S1 Fig — P. aeruginosa (NBRC 13275) incubated with various concentrations of lysozyme-chitosan oligosaccharide conjugates (LYZOX) solution in tryptic soy broth at 37°C for 3, 6, 9, 12 and 24 h. The dilutions were plated, the colonies were counted following growth overnight, and results were compared with the control. The values are the mean ± SEM from 11 independent experiments. Symbols: circles, LYZOX (10,000 μg/mL); squares, LYZOX (2,000 μg/mL); up-pointing triangles, LYZOX (1,000 μg/mL); down-pointing triangles, LYZOX (200 μg/mL). (TIFF) [file pone.0217504.s001.tiff]

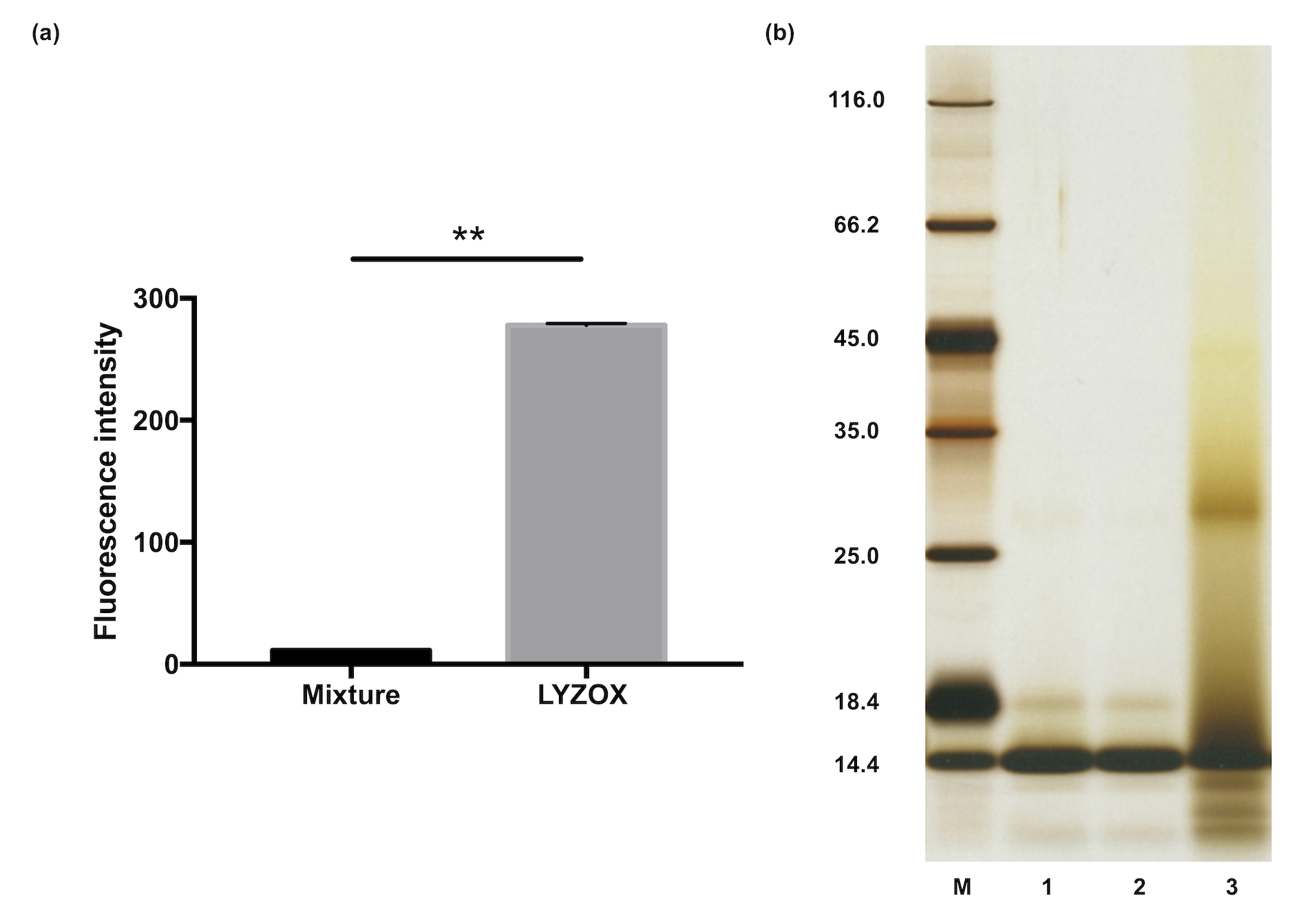

Supplement: S2 Fig — (a) Changes in fluorescence intensity produced by the Maillard reaction. The fluorescence intensity of the lysozyme-chitosan oligosaccharide conjugate (LYZOX) solution (500 μg/mL) and the mixture (lysozyme [250 μg/mL] and COS [250 μg/mL]) were measured (excitation: 370 nm/emission 440 nm). The values are the mean ± SEM of triplicate measurements. **p<0.01 (unpaired t-test). (b) Sodium dodecyl sulfate-polyacrylamide gel electrophoresis analysis with a 10–20% gradient gel. Ten microliters of LYZOX (500 μg/mL), lysozyme (250 μg/mL) or the mixture (lysozyme [250 μg/mL] and COS [250 μg/mL]) were loaded into each well. M, molecular weight marker; lane 1, lysozyme; lane 2, mixture (lysozyme and chitosan oligosaccharide); lane 3, lysozyme-chitosan oligosaccharide conjugate (LYZOX). (TIFF) [file pone.0217504.s002.tiff]

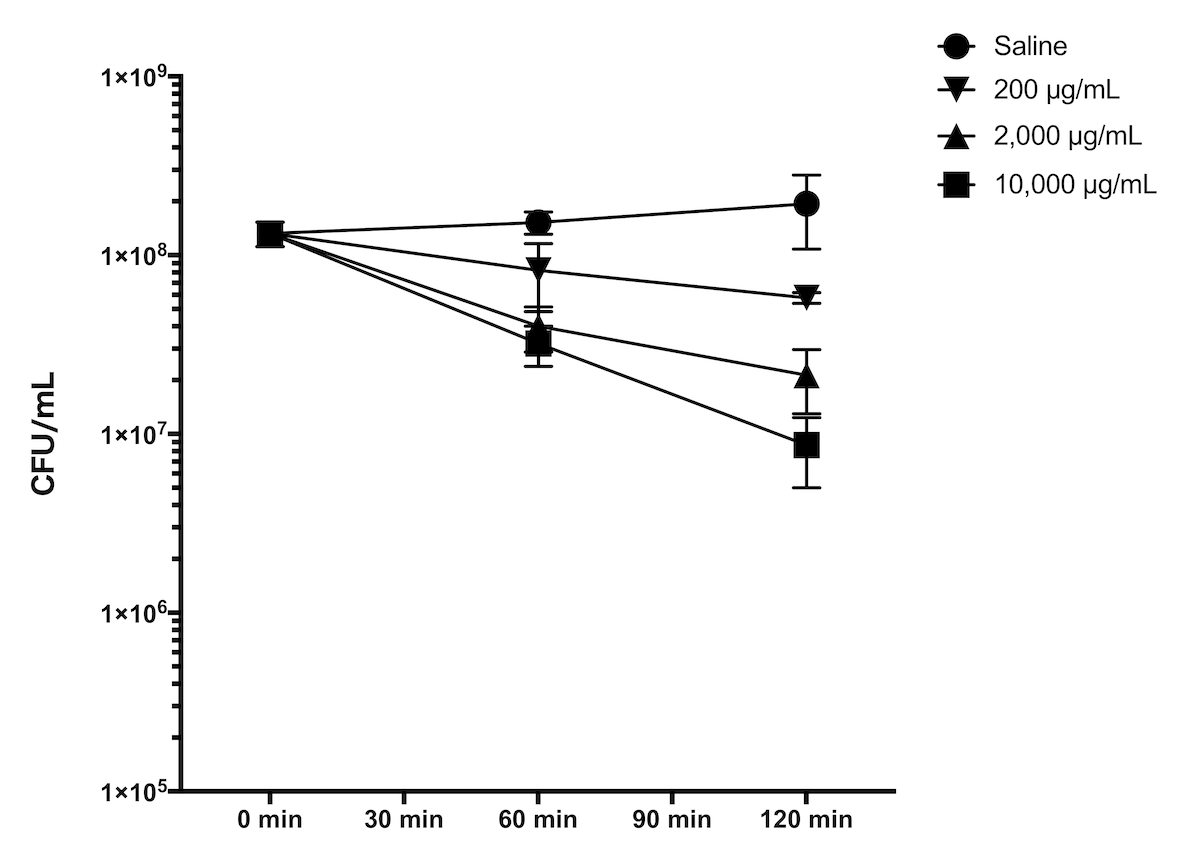

Supplement: S3 Fig — P. aeruginosa (NBRC 13275) was incubated with various concentrations of lysozyme-chitosan oligosaccharide conjugates (LYZOX) in saline at 37°C in a water bath for 0 min, 60 min and 120 min. The dilutions were plated, and the colonies were counted following growth overnight. The values are the mean ± SEM from three independent experiments. Symbols: circles, saline; down-pointing triangles, LYZOX (200 μg/mL); up-pointing triangles, LYZOX (2,000 μg/mL); squares, LYZOX (10,000 μg/mL). (TIFF) [file pone.0217504.s003.tiff]

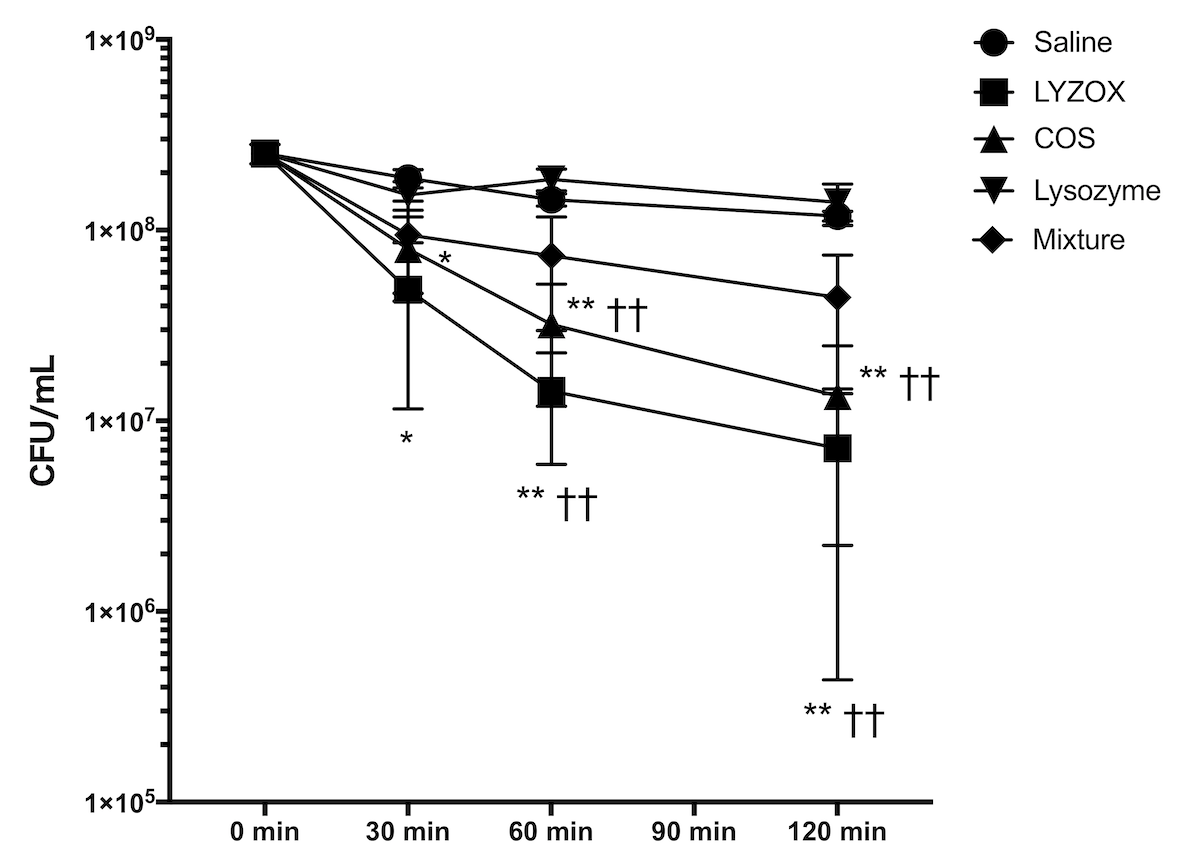

Supplement: S4 Fig — MRSA (IID 1677) was incubated with each treatment solution in saline at 37°C in a water bath for 0 min, 60 min and 120 min. Treatments were lysozyme-chitosan oligosaccharide conjugates (LYZOX) solution (2,000 μg/mL), chitosan oligosaccharide (COS) solution (1,000 μg/mL), lysozyme (1,000 μg/mL) and mixed solution (lysozyme [1,000 μg/mL] and COS [1,000 μg/mL]). The dilutions were plated, and the colonies were counted following growth overnight. The values are the mean ± SEM from four independent experiments. Symbols: circles, saline; squares, LYZOX; up-pointing triangles, COS; down-pointing triangles, lysozyme; rhombuses, mixture. *p<0.05 or **p<0.01 compared with saline; ††p<0.01 compared with lysozyme. (unpaired t-test). (TIFF) [file pone.0217504.s004.tiff]
