# Supplementary material for: Antibacterial activity of lysozyme-chitosan oligosaccharide conjugates (LYZOX) against Pseudomonas aeruginosa, Acinetobacter baumannii and Methicillin-resistant Staphylococcus aureus
Source: PLoS One. 2019 May 28;14(5):e0217504. doi: 10.1371/journal.pone.0217504 (PMC6538184; doi:10.1371/journal.pone.0217504)
Supplement: S1 Table — MIC: minimal inhibitory concentration. HMC: high molecular weight chitosan (molecular weight [MW] of 624 kDa). LMC: low molecular weight chitosan (MW of 107 kDa). CM: chitosan microparticles. HTCCs: N-(2-hydroxypropyl)-3-trimethylammonium chitosan chloride. HTCCs are water-soluble derivatives of chitosan (CS) that are synthesized by a reaction between glycidyl-trimethyl-ammonium chloride and CS. Six different polymers with different degrees of quaternization and different molecular weights were synthesized as HTTCs. N.D.: no data. Clinical isolate: CI. NDM: New Delhi metallo-beta lactamase. (DOCX) [file pone.0217504.s005.docx]

**S1 Table. Minimal inhibitory concentrations of chitosan or modified chitosan in previous reports.**

| Bacterial strain [ref] | | Chitosan or modified chitosan | MIC (μg/mL) |  |
| --- | --- | --- | --- | --- |
| Methicillin-resistant *Staphylococcus aureus* | CCUG 60578 [1] | HMC | 500 | |
|  | CI [1] | HMC | 500 | |
|  | CCUG 60578 [1] | LMC | 500 | |
|  | CI [1] | LMC | 500 | |
|  | N.D. [2] | CM | 1000 | |
|  | ATCC 33591[3] | HTCCs | 125 to 250 | |
|  | R3545, CI [3] | HTCCs | 125 to 250 | |
|  | R3889, CI [3] | HTCCs | 125 to 250≤ | |
| Vancomycin-resistant *Staphylococcus aureus* | ATCC 700699 [4] | HMC | 250 | |
|  | ATCC 700699 [4] | LMC | 250 | |
| Vancomycin-resistant *Enterococcus faecalis* | BAA 2365 [4] | HMC | 100 | |
|  | BAA 2365 [4] | LMC | 100 | |
| Vancomycin-resistant *Enterococcus faecium* | ATCC 51559 [3] | HTCCs | 125 to 250 | |
| Vancomycin-resistant *Enterococcus* | N.D. [2] | CM | 2000 | |
| Multidrug resistant *Acinetobacter baumannii* | CCUG 61012 [5] | HMC | 500 | |
|  | CI [5] | HMC | 1000 | |
|  | CCUG 61012 [5] | LMC | 750 | |
|  | CI [5] | LMC | 1000 | |
| NDM-1 producing *Acinetobacter baumannii* | R 676, CI [3] | HTCCs | 125 to 250 | |
| Multidrug resistant *Pseudomonas aeruginosa* | R 596, CI [3] | HTCCs | >250 | |
|  | R 590, CI [3] | HTCCs | ≥250 | |
| Extended-spectrum β-lactamase-producing *Escherichia coli* | N.D. [2] | CM | 2000 | |
| Multidrug resistant *Escherichia coli* | R 3597, CI [3] | HTCCs | 125 to 250 | |
|  | R 250, CI [3] | HTCCs | 250 to 250< | |
| Beta-lactam resistant *Klebsiella pneumoniae* | ATCC 700603 [3] | HTCCs | 250 to 1000 | |
| Multidrug resistant *Klebsiella pneumoniae* | R 3421, CI [3] | HTCCs | 125 to 500 | |
| NDM-1 producing multidrug resistant *Klebsiella pneumoniae* | R 3949, CI [3] | HTCCs | ≥250 | |
| NDM-1 producing *Enterobacter cloacae* | R 3921, CI [3] | HTCCs | 125 to 250 | |

MIC: minimal inhibitory concentration.

HMC: high molecular weight chitosan (molecular weight [MW] of 624 kDa).

LMC: low molecular weight chitosan (MW of 107 kDa).

CM: chitosan microparticles.

HTCCs: N-(2-hydroxypropyl)-3-trimethylammonium chitosan chloride. HTCCs are water-soluble derivatives of chitosan (CS) that are synthesized by a reaction between glycidyl-trimethyl-ammonium chloride and CS. Six different polymers with different degrees of quaternization and different molecular weights were synthesized as HTTCs.

N.D.: no data.

Clinical isolate: CI.

NDM: New Delhi metallo-beta lactamase.

**References**

[1] Costa EM, Silva S, Tavaria FK, Pintado MM. Insights into chitosan antibiofilm activity against methicillin-resistant Staphylococcus aureus. J Appl Microbiol. 2017;122(6):1547-57. Epub 2017/04/04. doi: 10.1111/jam.13457. PubMed PMID: 28370752.

[2] Ma Z, Kim D, Adesogan AT, Ko S, Galvao K, Jeong KC. Chitosan Microparticles Exert Broad-Spectrum Antimicrobial Activity against Antibiotic-Resistant Micro-organisms without Increasing Resistance. ACS Appl Mater Interfaces. 2016;8(17):10700-9. Epub 2016/04/09. doi: 10.1021/acsami.6b00894. PubMed PMID: 27057922.

[3] Hoque J, Adhikary U, Yadav V, Samaddar S, Konai MM, Prakash RG, et al. Chitosan Derivatives Active against Multidrug-Resistant Bacteria and Pathogenic Fungi: In Vivo Evaluation as Topical Antimicrobials. Mol Pharm. 2016;13(10):3578-89. Epub 2016/09/03. doi: 10.1021/acs.molpharmaceut.6b00764. PubMed PMID: 27589087.

[4] Costa EM, Silva S, Veiga M, Vicente S, Tavaria FK, Pintado ME. Investigation of chitosan's antibacterial activity against vancomycin resistant microorganisms and their biofilms. Carbohydr Polym. 2017;174:369-76. Epub 2017/08/20. doi: 10.1016/j.carbpol.2017.06.087. PubMed PMID: 28821080.

[5] Costa EM, Silva S, Vicente S, Veiga M, Tavaria F, Pintado MM. Chitosan as an effective inhibitor of multidrug resistant Acinetobacter baumannii. Carbohydr Polym. 2017;178:347-51. Epub 2017/10/21. doi: 10.1016/j.carbpol.2017.09.055. PubMed PMID: 29050604.
